# An AI-driven workflow for the accelerated optimization of cell-free protein synthesis

**DOI:** 10.1016/j.isci.2025.113599

**Published:** 2025-09-19

**Authors:** Mostafa M. Khalil, Aisha Elsawah, An N. Hoang, Jean-Loup Faulon, Baptiste Panthu, Joan Hérisson

**Affiliations:** 1 Université Paris-Saclay, INRAE, AgroParisTech, Micalis Institute, 78352 Jouy-en-Josas, France; 2 Manchester Institute of Biotechnology, SYNBIOCHEM Center, School of Chemistry, The University of Manchester, Manchester M1 7DN, UK; 3 Genomics Metabolics, Genoscope, François Jacob Institute, CEA, CNRS, University Evry, Université Paris-Saclay, 91057 Evry, France; 4 Université Claude Bernard Lyon1, CarMeN Laboratory, INSERM, INRAE, 69310 Pierre-Bénite, France

**Keywords:** protein, metabolic engineering, synthetic biology

## Abstract

Cell-free protein synthesis (CFPS) is a versatile tool for rapid biological prototyping. However, exploring the large number of component combinations is a very time-consuming process. Active learning (AL) is known to reduce the number of experiments required, but is rarely integrated into routine laboratory workflows. To address this, we developed a fully automated Design-Build-Test-Learn (DBTL) pipeline that streamlines this optimization process with an improved AL strategy that selects informative and diverse experimental conditions. The Design phase was created entirely using ChatGPT-4 without manual code revisions, dramatically reducing coding time. This pipeline was implemented in a modular way within the Galaxy platform, following the Findable-Accessible-Interoperable-Reusable (FAIR) principles. When applied to the optimization of colicin M and E1 in both Escherichia coli and HeLa-based CFPS systems, a 2- to 9-fold increase in yield was achieved in just four cycles. This framework enables reliable, automated workflows for routine synthetic biology.

## Highlights

• ChatGPT-4 generated code for experimental design, no edits needed
• Active learning with a cluster margin approach needs less to learn more
• Colicins have been produced with up to a 9-fold yield improvement
• Colicins produced retained strong antimicrobial activity

## Graphical abstract

**Figure:**
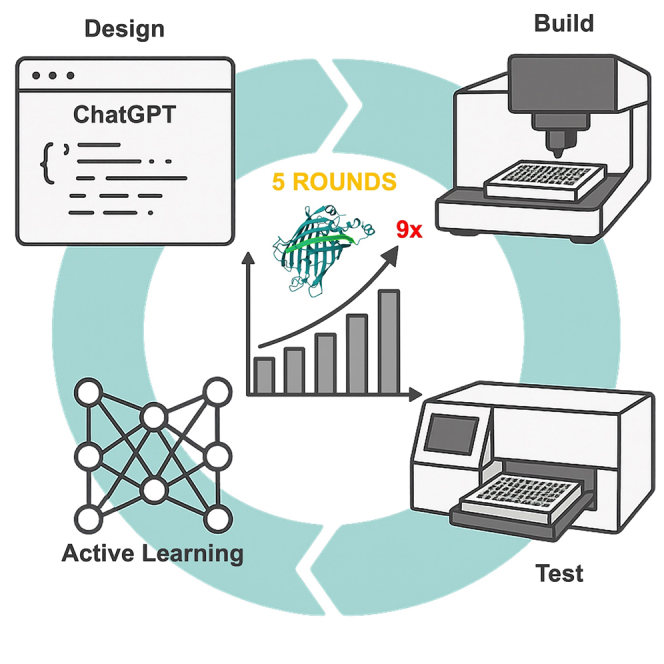

## Introduction

Proteins are naturally produced in all cells to perform a wide range of biological functions. Controlling their production can meet needs in several areas of society, including health, environment, energy, and food. Since the advent of genetic engineering some sixty years ago, it has become relatively easy to modify a living cell to produce a desired protein. However, there are significant challenges associated with cellular production, particularly in terms of cell integrity. It is possible to overcome these cellular limitations by using minimal cell-free systems^1^ that contain only the essential components needed for protein production. For synthetic biology, the cell-free approach represents a renewed interest^2^^,^^3^^,^^4^ and the global market for cell-free protein synthesis (CFPS), or cell-free protein expression (CFPE) systems, has experienced a significant expansion over the past decade, with projections for a similar trajectory over the next decade.^5^^,^^6^ The industrialization of these cell-free systems would go a long way toward meeting the challenges of today and tomorrow, and CFPS systems are now well established as robust platforms for the synthesis of a wide variety of compounds.^7^^,^^8^^,^^9^^,^^10^ The increasing availability of such commercialized systems and substrates makes it easier to make a CFPS platform accessible to any research laboratory. However, its widespread adoption is hampered by the need to further develop various tools to streamline and automate the process. This includes the ability to accurately assess protein yields, select an appropriate acellular matrix, and optimize the assembly of CFPS components altogether, taking into account the prokaryotic or eukaryotic origin of the cell-free system.^7^^,^^10^^,^^11^^,^^12^^,^^13^ Given the urgent need for novel antibacterial strategies, we selected colicins as model antimicrobial proteins to demonstrate the capabilities of our CFPS platform, using their production as a proof of concept relevant to the development of next-generation antimicrobial therapies. This selection was driven by several key considerations: (i) colicins are increasingly considered as promising alternatives to conventional antibiotics, mainly due to their specificity and lack of toxicity to human cells—their cytotoxic activity is restricted to bacteria that express specific receptor proteins^14^; (ii) certain colicins—specifically Colicin M, and E1—have already been produced in Escherichia coli-based CFPS systems^15^^,^^16^^,^^17^; (iii) colicins have a modular domain structure, which offers flexibility for protein engineering. This property enables the design of novel colicin variants with potentially enhanced or tailored antimicrobial functionalities, aligning well with the goals of synthetic biology and cell-free bioproduction platforms. Moreover, the choice of both Colicin M (∼271 amino acids, ∼29 kDa) and Colicin E1 (∼522 amino acids, ∼58 kDa) enables the evaluation of the CFPS platform’s performance across a range of protein sizes, which is important because protein size significantly impacts transcription, translation, and folding efficiency in cell-free systems.

In addition to advances in cell-free techniques, synthetic biology has seen the automation of its methods within dedicated platforms. These platforms, which are not limited to specific equipment, bring together in a single location all the resources needed to carry out a project. In 2019, a worldwide initiative launched the Global Biofoundries Alliance, which currently includes more than 30 academic biofoundries around the world.^18^ Biofoundries are dedicated to advancing and accelerating research in engineering and synthetic biology for both academic and practical applications. They support this using automation, high-throughput technologies, process up-scaling, computational design tools, and innovative methodologies. Here, we question the position of artificial intelligence (AI) as a computational cornerstone of such platforms. The use of iterative design-build-test-learn (DBTL) cycles in biological engineering should enable researchers to evaluate large genetic constructs, and the integration of AI and machine learning (ML) approaches can be leveraged to refine the design processes. However, there are relatively few studies that have performed the full DBTL cycle on a real case, although some open-source software tools are available for this purpose.^19^^,^^20^^,^^21^ Today, most biofoundries have intensively developed the build and test steps with a large set of equipment and a good level of automation. Many manufacturers offer integrated solutions that can handle equipment related to the build step (e.g., liquid handlers) as well as those related to the test step (e.g., plate readers) through programmable features (workflows and scheduling) and robotic arms. This area is already well developed, and there are no major advances currently anticipated. However, while all biofoundries use some software in their design step, this is poorly automated, forcing users to convert data from one software tool to make it compatible with another. In addition, few use machine learning techniques, either within the tools or through the Learn step. Moreover, the software developed in biofoundries is very sophisticated and is the result of several months of work by experts in both programming and specific bioengineering applications. Writing such code is therefore beyond the reach of most scientists who are not computer literate. Although several works on cell-free automation and coding capabilities of liquid handlers have been conducted in recent years,^22^^,^^23^ further efforts are needed to develop an integrated cell-free platform with fully automated liquid handler encoding features.

One of the main challenges in establishing an efficient cell-free system lies in identifying the optimal composition of components within the cell extract. This process requires in-depth knowledge of both the cell-free platform and its molecular constituents and can be time consuming and costly. While cell-free systems adopt a reductionist approach, they still encounter the inherent complexity of biological processes. Although many CFPS platforms are robust and capable of producing a variety of proteins under standard conditions, the limited availability of public data on the critical components' contribution means that further optimization is often required to improve production yields, particularly in system-specific contexts or for more complex protein targets. To address this challenge, various active learning (AL) frameworks have been applied to identify “globally optimal” compositions with relatively few experimental iterations.^24^^,^^25^ AL is a machine learning approach in which the model selectively queries the most informative data points for labeling to efficiently improve its predictive performance while minimizing the number of required experiments.^26^

One of the most important aspects of the AL process is determining how much new data to be labeled before retraining the model.^27^ Classical AL methods often operate in a sequential manner, selecting and labeling a single data point at a time. However, retraining the model after each sample can be inefficient, particularly when the time required to retrain the model may not be negligible, or the addition of a single sample to the training set may not have a meaningful impact on model performance, especially for deep learning models.^28^ Therefore, in situations where multiple experiments are run in parallel, batch AL has been proposed as a solution that allows multiple data points to be selected and labeled before retraining the model.^29^ However, sampling based on uncertainty alone often leads to bias, where the currently selected sample is not representative of the distribution of unlabeled datasets, and on the other hand, focusing solely on sample diversity can increase labeling costs, as it may result in the selection of multiple samples with low information content.^30^ In batch AL, it is important that the selection criteria take into account both the diversity of the samples and the amount of information provided by each sample relative to the model.^31^ A simple and easy to implement strategy that considers both diversity and uncertainty of new data has been proposed, called Cluster Margin (CM).^32^

Large language models (LLMs) have revolutionized natural language processing (NLP) and shown significant potential in automating code generation. These models leverage vast amounts of textual data to understand and generate human-like text, including programming code or machine-readable protocols.^33^ There are several tools that exist to generate code from NLP, among the most famous ones we can cite ChatGPT, GitHub Copilot, and Visual Studio Code. However, in most cases, the code must be corrected or tuned by the programmer to run as expected, sending back to the need of an expertise in computer language. Beyond the code itself, LLMs can be also used to understand already written code^34^ or generate high-quality test sets.^35^ Recently, ChatGPT has even been used to design, plan, and perform complex chemical synthesis experiments^36^ such as browse the web, access documentation, control robotic systems, and collaborate with other LLMs to carry out six key tasks: planning chemical syntheses, navigating hardware documentation, executing cloud lab commands, controlling liquid handlers, managing complex multi-module tasks, and solving optimization problems using experimental data. This opens a door that needs to be consolidated and extended.

In this article, we present a modular, fully automated DBTL workflow for optimizing CFPS in both bacterial and mammalian systems. The pipeline integrates all stages of the DBTL cycle—ranging from experimental design and microplate layout generation to liquid handling execution, readout calibration, and data-driven selection of new experimental candidates—within the Galaxy platform, ensuring FAIR compliance, reproducibility, and accessibility for non-programmers. A key feature of the workflow is an improved AL strategy based on CM sampling, which prioritizes experiments that are both uncertain and diverse, thereby accelerating optimization with fewer experimental iterations. As a case study, we applied this platform to optimize the expression of the antimicrobial proteins colicin M and colicin E1 in *E. coli* and HeLa-based CFPS systems, using software components generated entirely by ChatGPT-4 from non-specialist prompts, without manual code editing. Overall, this platform provides a practical foundation for next-generation biofoundries, integrating molecular biology, automation, and AI to streamline synthetic biology research and biomanufacturing.

## Results

### Overview of the cell-free protein synthesis system workflow

**Figure 1:**
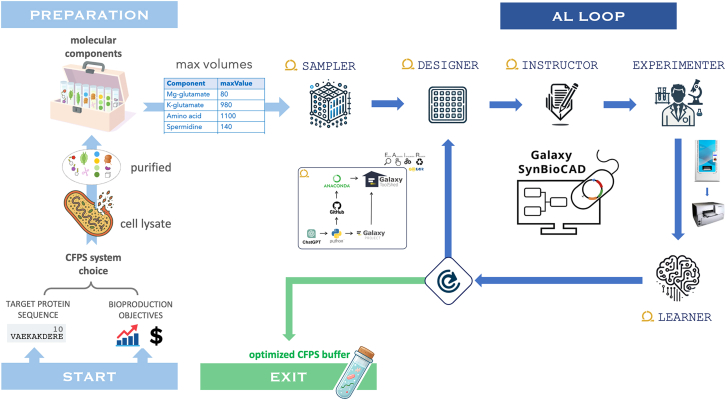
Overview of the CFPS system workflow To get started, the user chooses the CFPS system that matches the bioproduction objectives (increase yield, reduce price…). After cell lysate preparation, a molecular toolbox is obtained with different components and associated possible volumes for each of them. Next, the AL loop searches for a mixture with the highest yield by selecting and experimenting with different cell-free recipes, then re-learning from its newly acquired data and suggesting the next experiments to be tested. The loop starts by running the Sampler module once, generating an initial set of volume combinations using LHS, and outputting a CSV file with these samples. The Designer module then converts these samples into source and target microplate representations, producing two CSV files detailing the component volumes and feeding the Instructor, who writes the instructions for an ECHO 650 liquid handler. Both the source plate and the instruction file are used by the experimenter via the liquid handler, and the final microplate is analyzed using a BioTek Synergy HTX multimode microplate reader. If the AL process is not completed, the expression levels are analyzed and calibrated before feeding the Learner module, which provides new samples for testing. Finally, if the yield cannot be further improved, the user is provided with the optimal combination of the cell-free components.

Our automated workflow, shown in Figure 1, implements each step of the DBTL schema to optimize the production of the antimicrobial proteins, colicin M and colicin E1, in both *E. coli*- and HeLa-based CFPS systems. All Python scripts deployed within the Design stage were generated by ChatGPT-4, with no further manual editing. The process begins with the Sampler module that takes the maximum volume for each component in the CFPS reaction and generates initial experimental combinations via Latin-Hypercube Sampling (LHS), which ensures that the solution space is well represented. Then, from a set of combinations to be tested, the Designer generates source and destination microplate layouts that specify the location and volume of each component. These layouts are converted by the Instructor module into well-specific instructions compatible with automated liquid handlers. This step ensures fidelity between computational design and physical implementation. Together, these modules provide a modular and fully reproducible approach for defining and preparing the experimental space in a way that is compatible with downstream automation and learning. The Build and Test stages are addressed by the Experimenter module, which involves the physical implementation of the designed experiments using laboratory automation. Liquid handling is carried out by the Echo 650 acoustic dispenser, which transfers nanoliter volumes of CFPS components into 384-well plates according to the instructions generated by the Instructor module. After dispensing, the plates are sealed and incubated under appropriate conditions. Upon the completion of the reaction, fluorescence measurements are taken using a multi-mode plate reader to quantify protein expression levels. Then, the Learner module ensures the Learn stage by analyzing experimental outcomes and selecting the next set of combinations using CM sampling. By iterating through multiple DBTL cycles, the system refines its model and progressively identifies higher-yielding formulations. At the end of this process, which is marked by a substantial increase in production, the user is equipped with an optimized cell-free buffer tailored for the efficient synthesis of the desired protein.

### Detecting and quantifying cell-free synthesized proteins

**Figure 2:**
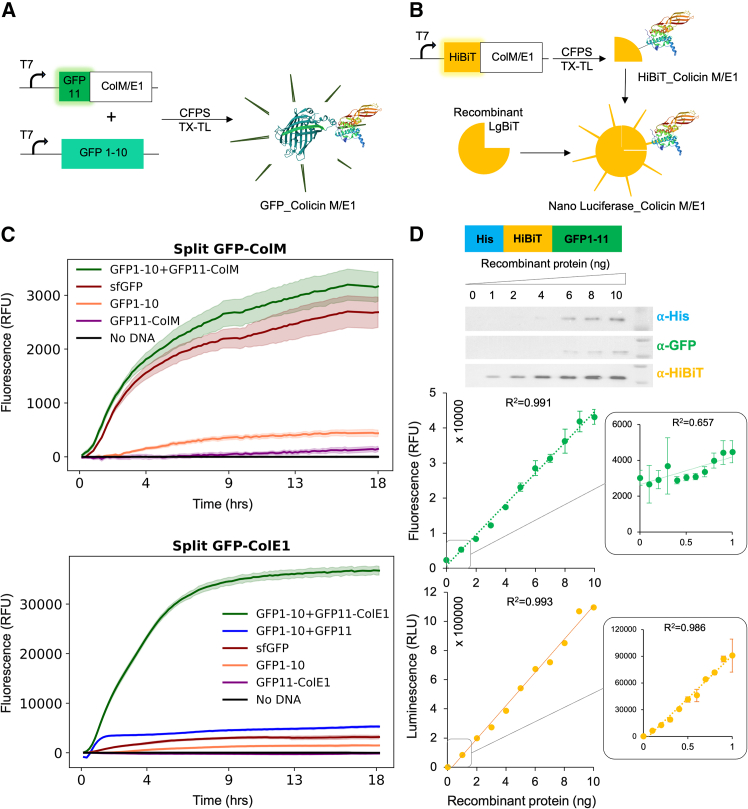
Detecting and quantifying cell-free synthesized proteins (A) Split-GFP tag consists of GFP α-helix 11 as a tag fused to any of the colicins. Co-expression of truncated GFP1-10 and GFP11 tagged with colicins in a transcription-translation (TX-TL) coupled CFPS system restores fluorescent GFP. (B) Split-Nanoluciferase consists of the HiBiT domain as a tag fused to the colicin. After the TX-TL CFPS reaction, the recombinant large subunit of the luciferase (LgBiT) is added together with luciferase substrate to assemble a functional Nanoluciferase. (C) Kinetics of fluorescence detection after the co-expression of the gene encoding GFP1-10 and GFP11 with colicins (dark green), or single expression of GFP1-10 (coral) or GFP11 (purple). Positive controls were sfGFP (dark red) and the combined GFP1-10 and GFP11 (blue). Both assays were performed in 10.5 μL reactions at 30°C, with all the fluorescence readings normalized to the No-DNA control. All plasmid DNA final concentrations were 6 nM for the split-GFP-ColM experiment and all DNA were 5 nM for the split-GFP-ColE1 experiment. (D) Data are presented as means ± SD for six independent experiments (*n* = 6) using a unique batch of recombinant his-HiBit-GFP purified proteins. Design and purification of the His-HiBiT-GFP1-11 to compare fluorescence and luminescence sensitivity thresholds. The protein was purified using the His tag and quantified using the Bradford method. A range from 1 to 10 ng of recombinant protein was detected by Western blot (top panel) using His antibody (α-His), GFP antibody (α-GFP) or HiBiT antibody (α-HiBiT). The untrimmed gel is shown in Figure S4. A range of 1–10 ng then 0.1 to 1 ng of His-HiBiT-GFP1-11 was quantified by fluorescence (green) and luminescence (orange). Readings were measured from the same well deposit with 6 replicates in a final volume of 2 μL for fluorescence, then 2 μL of substrate and LgBiT were added for luminescence. The Pearson correlation coefficient (*R*^2^) was used to determine the sensitivity threshold for both fluorescence and luminescence.

To demonstrate the versatility of our workflow beyond the optimization of a single protein in a single cell-free system, colicins M and E1 were produced in a prokaryotic CFPS (proCFPS), and colicin M was selected to be produced in an eukaryotic CFPS (euCFPS). Prior to applying the workflow, we tested the production and quantification of recombinant colicins by designing two different detection methods to validate our genetic constructs: a split green fluorescent protein (GFP) fluorescence system (Figure 2A) and a luminescent HiBiT system (Figure 2B). A His tag (consisting of 6 consecutive histidines) was integrated in both systems to enable protein purification for downstream applications in the biofoundry. We constructed a split-GFP system based on a GFP11 tag fused to any of the colicins,^37^ and co-expressed with the GFP1-10 fragment (Figure 2A). The ability of the split-GFP constructs to show fluorescent activity only upon the co-expression of both *GFP1-10* and *GFP11* was tested in proCFPS. The reassembled constructs of the two plasmid DNAs for *ColM* or *ColE1* showed a rapid increase in fluorescence (Figure 2C) over 18 h, reaching a plateau at approximately 10 h, indicating a highly efficient expression. Full-length superfolder GFP (*sfGFP)* was used as a positive control. GFP1-10 alone showed a very low level of fluorescence, significantly lower than the fully assembled GFP construct. The *GFP11-ColM* and *GFP11-ColE1* fragments, as well as the No DNA sample, showed minimal or nearly undetectable fluorescence. The significant difference in fluorescence observed between the colicin E1 and colicin M systems is attributed to their solubility and stability characteristics. Colicin M fused to split GFP is more prone to aggregation, leading to reduced stability and increased degradation, which results in an overall low fluorescence signal. In contrast, colicin E1 is more soluble and properly folded in extracellular conditions, yielding a substantially higher fluorescence.^17^^,^^38^^,^^39^ This effect is obvious in our data, where the full GFP_ColE1 construct exhibits nearly 10-fold greater fluorescence levels than that of colicin M.

We constructed the luminescent system based on a HiBiT tag placed at the N-terminal part of our recombinant colicins, as suggested by the supplier, that would be assembled to a large bit LgBiT for luminescence detection upon the substrate addition (Figure 2B). In addition, we added the His tag upstream of the HiBiT tag due to the high sensitivity of the HiBiT tag (Figure 2D). In a primary set of experiments to test the position of the double His and HiBiT tags at the N-terminus of sfGFP, we observed that fluorescence and luminescence measurements were independent of the position of the tag, which had very little effect (Figure S1). Purified recombinant HiBiT-sfGFP protein was then used to determine the sensitivity thresholds for both luminescence and fluorescence detection (Figure 2D). A wide range of protein concentrations from 0 to 10 ng of purified recombinant HiBiT-GFP in 10 μL showed a linear luminescence and fluorescence signal. A well-defined linearity was observed between 0 and 1 ng of protein for luminescence only, but not for fluorescence. The use of a highly reproducible and low-volume automated platform (Figure S2) highlighted the ability to obtain a linear quantification of fluorescence and luminescence activity in 2 μL of reaction, but not in 1 μL (Figure S3). We have shown that the strength of using luminescence is its ability to quantify small amounts of proteins, whereas the strength of fluorescence is to allow real-time protein synthesis expression, in contrast to the luminescent-based assay, which requires the addition of a substrate at each time point, which interrupts the automatic kinetics acquisition. Based on these findings, our choice was to use the split-GFP system for proCFPS optimization, while the HiBiT luminescent system in a final volume of 2 μL for euCFPS, as it is already known that the respective yields of euCFPS are low compared to proCFPS.^7^

### Optimizing cell-free protein synthesis systems' productivity

To evaluate the productivity of our proCFPS system, we simplified the reaction formulation by excluding several non-essential components—namely tRNAs, cAMP, NAD, CoA, and folinic acid—as shown in a previous study.^24^ This allowed us to focus on nine key components, treated as parameters, whose concentrations were systematically varied. However, the other two core components—namely *E. coli* cell-free lysate and HEPES buffer—were kept constant (see STAR Methods section). Colicin M production was assessed using six concentration levels for each of the nine variable parameters (0%, 25%, 50%, 75%, 100%, and 125%), where 100% corresponds to the standard buffer composition^40^ which lies within the guidelines of the commercial provider of the *E. coli* lysate. This setup resulted in a combinatorial space of 6^9^ > 10 million possible compositions. For colicin E1, the nine parameters were varied by fixed incremental steps of 20 nL from their respective maximum volumes, corresponding to 125% of the standard buffer, leading to a combinatorial space of approximately 4 trillion possible compositions (see STAR Methods and Table S1 for details). In the case of the commercial euCFPS system used, we defined our reference condition as 0.5X of the standard buffer composition, using half the volume of each of the four components specified by the supplier. This decision was due to the liquid handling constraints when transferring highly concentrated stock DNA solutions for a final 2 μL reaction (see STAR Methods). We applied incremental steps of 20 nL to define the volume range for each component. The maximum volumes representing 0.925X of the standard composition for the HeLa lysate, accessory proteins, reaction mix, and DNA were 940 nL, 200 nL, 380 nL, and 480 nL, respectively. This strategy resulted in a combinatorial space of over 214,000 possible compositions, calculated by dividing each maximum volume by 20 nL and multiplying the resulting values.

The pipeline starts with the conversion of the maximum concentrations of each of the nine components of proCFPS and the four components of euCFPS into their corresponding volumes. The resulting file was then processed using the Galaxy-SynBioCAD workflow *AI-CellFree – Init* (Figure S5), which integrates the Sampler, plate Designer, and Instructor modules, resulting in the generation of ECHO instruction files. After completing the first loop, the resulting fluorescence or luminescence data were used as input for the workflow *AI-CellFree – Core* (Figure S6), which includes the Learner, plate Designer, and Instructor modules. This was run as an iterative process (Figure 1). The final output of the previous modules in each iterative workflow is a new ECHO instruction file(s), which contain the precise volumes to be transferred from a specific source well to a designated destination well that is loaded into the ECHO “Cherry Pick”’ software. As transferring all the components using the ECHO liquid handler takes more than 3 h to reach the final volume for proCFPS, we transferred 9 out of 11 parameters by ECHO, leaving the remaining fixed volume of 4 μL for lysate and water to be manually pipetted into the destination plate for efficiency and time-saving. Following this, the destination plate was loaded into a microplate reader to measure colicin production levels in each well. As there is quite high variability in cell-free systems, a calibration process (see STAR Methods section) was required to calibrate the luminescence or fluorescence levels of one plate in relation to the others.

**Figure 3:**
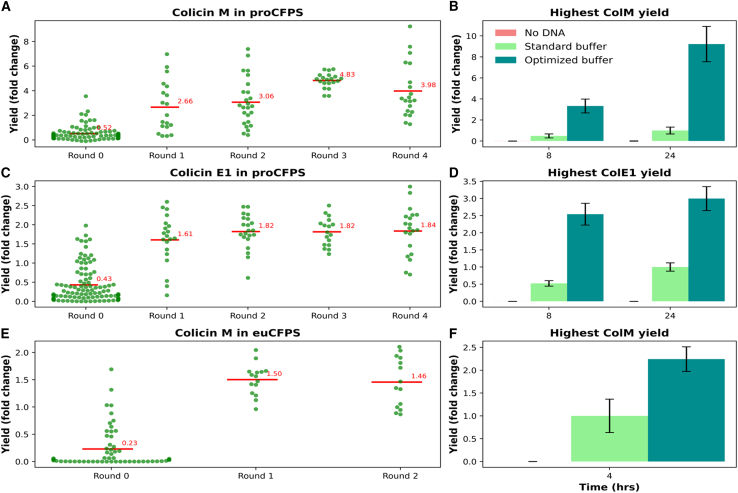
Optimizing CFPS systems productivity (A–F) The figure shows the production of colicin M (A) and (B) and colicin E1 (C) and (D) in the proCFPS system, and colicin M in the euCFPS system (E) and (F). The swarm plots (A), (C), and (E) compare the yields obtained over several AL test loops using CM strategies. Each green dot in any round represents the mean of one experiment through 3 repetitions, based on a unique buffer composition for the compositions mix of this specific round. Each round (or loop) shows the average yields across all the combinations marked by the horizontal line in red. Each column with error bar of the graphs (B), (D), and (F) presents mean ± SD for 3 repetitions of the same experiment. Graphs (A) and (B) show the distribution and variability of the yields for each round and illustrate a very clear improvement in the colicin M production of up to 9-folds in our proCFPS system, while a successful improvement of 3-folds for colicin E1 was achieved in proCFPS (C). In the euCFPS system, a doubling of the yield for the standard buffer (E) was ensured. Graphs (B) and (D) show the maximum yield fold change of the best predicted buffer composition of all rounds, and is compared to the standard buffer composition over two time points (8 and 24 h) for both colicin M and E1 in proCFPS. While (F) shows the maximum luminescence yield achieved at a single reading point at 4 h.

For each system, the model input consists of the concentration or the corresponding volume of all fluctuating components in each cell-free mix (Table S1). The ratio of each component to its maximum concentration is a critical factor; therefore, both concentration and volume values are normalized before being used for the model training. The model output is either the yield in colM and colE1 in the proCFPS system or the luminescence value for colicin M in the euCFPS system. Our Learner module is capable of handling both types of output seamlessly. The calibrated data were then used to incrementally train the model. The CM method was used to select the data for training more efficiently, with the aim of achieving the same performance as our vanilla method (see STAR Methods section). In each loop, the Learner module used labeled data from previous loops to train the model and then provided new samples to start the next loop. Figure 3 shows that up to a 9-fold increase in the yield of colicin M in proCFPS was achieved (Figure 3A), a 3-fold increase in the yield of colicin E1 in proCFPS (Figure 3C) was successfully achieved despite its inherently high fluorescence compared to colicin M, all compared to the yield of the standard buffer found in the literature.^40^ A 2-fold increase in colicin M yield was achieved in the euCFPS system (Figure 3E), which is promising as the optimization was performed using a well-characterized commercial HeLa lysate system. The newly optimized buffer composition maintained the performance as the supplier’s standard buffer (see STAR Methods section), while using reduced volumes of all components, thereby lowering the overall reagent costs.

In Loop 0, component combinations were generated using the Latin Hypercube Sampling (LHS) method from the Sampler module. At this early stage, low yields were predominantly observed (Figures 3A, 3C, and 3E), largely due to the random nature of the initial sampling. Since the model had not yet been trained on meaningful data, its performance was limited, and it tended to favor exploration to improve predictive accuracy. Occasionally, a higher-yielding combination was identified by chance. As the optimization loops progressed, both the yield outputs and the model performance improved (Figure S14), reflecting an enhancement in the overall effectiveness of the cell-free system. While some highest-yielding combinations (Table S4) were discovered in Loop 4 for proCFPS systems and in Loop 2 for the euCFPS system, it is possible that the global optimum had not yet been reached. Nonetheless, we chose to conclude the experiments at this point, as the yield increase achieved was already satisfactory and sufficient to demonstrate the effectiveness of our LEARNER module.

### Measuring synthesized proteins activity

The purified recombinant HiBiT-GFP protein was used to determine the detection threshold of both luminescence and fluorescence (Figure 2D) by generating a range of concentrations to relate the readings to the amount of a given protein. This allowed us to quantify the level of colicin expression in either pro- or euCFPS. A strong linear correlation between luminescence or fluorescence signals and known protein amounts was observed, which enabled the conversion of CFPS reaction volumes into estimated nanograms of expressed colicin, to be used for the antimicrobial activity assay.

**Figure 4:**
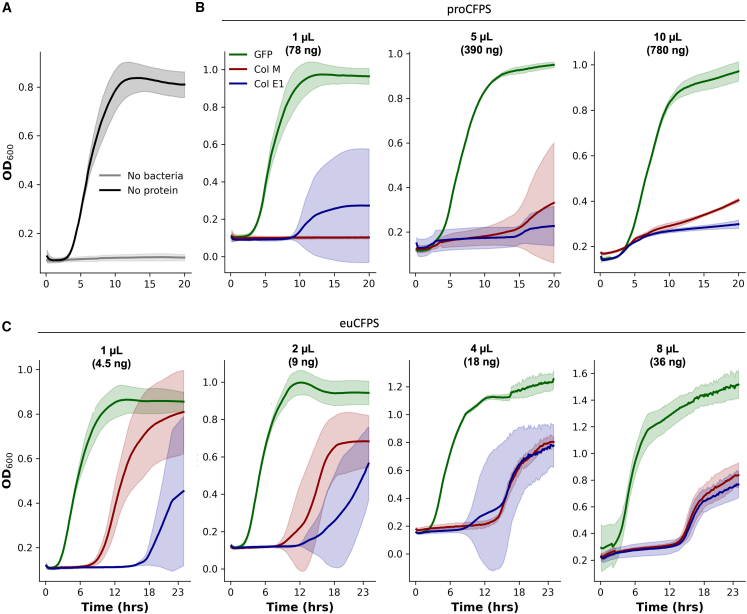
Measuring synthetized proteins activity (A–C) Bacterial growth of the *E. coli W3110* strain was monitored over 20 h by optical density measurement at a wavelength of 600 nm in (A) the absence of recombinant protein (B) or in the presence of non-purified GFP (green), colicin M (red) and colicin E1 (violet) produced in the *E. coli* commercial lysate of proCFPS and in (C) commercial euCFPS system based on HeLa lysate. Each bacterial growth assay was performed in a final volume of 50 μL, supplemented with 1, 5, or 10 μL of proCFPS containing neosynthesized colicin, corresponding to 78, 390, or 780 ng of protein, respectively, or with 1, 2, 4, or 8 μL of euCFPS containing neosynthesized colicin, corresponding to 4.5, 9, 18, or 36 ng, respectively. The error bar indicates the variability of three replicates of colicin produced in three separate CFPS reactions.

Bacterial growth measurements over time showed a strong inhibitory effect of both colicins (Figure 4). In proCFPS, this effect was observed with 1, 5, or 10 μL of reaction mix containing colicins, corresponding to 78, 390, or 780 ng of colicin per 50 μL of bacterial culture. In euCFPS, a dose-dependent inhibition was observed with the addition of 1, 2, 4, or 8 μL, corresponding to 4.5, 9, 18, or 36 ng of colicin. Colicins M and E1 showed no significant difference in their antimicrobial activity with respect to protein concentration after production in proCFPS. However, in euCFPS, colicin E1 showed strong antimicrobial activity, retarding bacterial growth for more than 15 h, even at the lowest tested level of 4.5 ng per 50 μL of culture. In contrast, colicin M was most effective at 18 and 36 ng (i.e., 0.36 and 0.72 ng/μL, respectively), while lower concentrations only delayed exponential growth to 12 h. These results show that protein levels correlate with functional activity and that even low-yielding systems such as euCFPS produce sufficient and active forms of colicins.

## Discussion

This work presents a fully automated, modular, and reproducible DBTL workflow that broadens the current horizon of cell-free biofoundries. By integrating all stages of the DBTL cycle into the Galaxy scientific workflow manager, and using a code fully generated by ChatGPT-4 for the Design phase, we have demonstrated that non-specialists can quickly configure and run complex CFPS optimization experiments. This workflow is also being used to operate the Cell-Free Biofoundry at the Paris Biofoundry.

Another key innovation is our implementation of a CM-based AL strategy that improves traditional AL methods by balancing uncertainty and diversity in sample selection. This strategy allowed the exploration of a vast combinatorial space—spanning from millions to trillions of possible buffer compositions—while converging on high-performance combinations in just four iterations. The ability to achieve up to 9-fold improvements in the yield of colicin M, 3-fold increase in a high inherently fluorescent colicin E1 in proCFPS, and 2-fold improvements in an well optimized commercial euCFPS platform highlights the generalizability and robustness of the pipeline.

In addition, we validated two detection systems (split-GFP and HiBiT luminescence) to assess CFPS productivity across platforms. These systems allowed us to track kinetic expression and quantify final protein yields. We also demonstrated that synthesized colicins retain antimicrobial activity in bacterial growth assays. This functional validation is critical to demonstrate not only the production efficiency but also the biological relevance of the synthesized proteins.

The use of LLM-generated code for the Design phases represents a shift in how pieces of code can be designed and deployed. While AI-assisted code writing is common, our study shows that entire experimental pipelines, including robot control and layout generation, can be fully coded and deployed without any further manual editing. This opens new possibilities for reproducible and user-friendly deployment of such software platforms.

An important potential development is the integration of additional types of liquid handlers, beyond the ECHO system used in this study, and the incorporation of robotic arms to automate more of the experimental process. Handling a variety of liquid handlers, including more common and accessible models, would increase the applicability of the workflow across different laboratories and platforms. By supporting a wider range of liquid handlers, the system could adapt more flexibly to different experimental setups. By introducing robotic arms for tasks such as sample preparation, microplate manipulation, and loading/unloading of liquid handlers and plate readers, the workflow could achieve a higher degree of automation, minimizing human intervention and helping to reduce experimental variability and handling errors, and speeding up the optimization process, making the system more scalable for high-throughput applications.

As AI continues to evolve, we anticipate that future developments will lead to even more efficient, scalable, and autonomous systems, driving advances in biomanufacturing and other areas of biotechnology.

### Limitations of the study

Although our workflow demonstrates acceleration and yield optimization in CFPS, we recognize that these platforms differ fundamentally from live-cell engineering. The modular and acellular nature of CFPS allows for controlled experimentation that closely resembles the optimization of chemical reactions. Consequently, the current implementation of the workflow is most appropriate for use cases involving rapid prototyping, enzyme production, or functional assays that can be performed *in vitro*. That said, bridging the gap between cell-free and *in vivo* systems remains an area of interest for future development. For instance, workflow extensions could entail coupling CFPS outputs with metabolic burden modeling or integrating data into iterative design loops for cell-based chassis.

Another limitation inherent in cell-free systems is their variability, such as fluctuations in component activity and lysate instability, which requires careful calibration and standardization across experiments. In addition, some of this variability may be due to the complexity of using two separate DNA constructs that must assemble to form a functional GFP protein. Variations in the folding or interaction dynamics between the expressed proteins may affect how well the constructs align and assemble, leading to differences in reassembly between replicates. Moreover, the luminescent-based assay requires substrate addition at each time point, which limits real-time monitoring and may introduce further variability. Future work could focus on further automating wet lab steps, including sample preparation and data collection, to minimize human intervention and reduce experimental variability. In addition, while our model converged to a very good solution within a few iterations, there may be potential to improve accuracy and yield further results with additional iterations or by refining the sampling strategy used in the initial phase.

## Resource availability

### Lead contact

Further information and requests for resources and reagents should be directed to and will be fulfilled by the lead contact, Joan Hérisson (joan.herisson@univ-evry.fr).

### Materials availability

The fully annotated DNA constructs generated in this study have been deposited at Zenodo at https://doi.org/10.5281/zenodo.14902238 and is publicly available as of the date of publication.

### Data and code availability

• All data generated and used during the Active Learning process in this study have been deposited at Zenodo at https://doi.org/10.5281/zenodo.14904992 and are publicly available as of the date of publication.
• All original code has been deposited at Zenodo at https://doi.org/10.5281/zenodo.14900104 and is publicly available at https://github.com/brsynth/icfree-ml as of the date of publication.
• All Conda packages have been deposited at Zenodo at https://doi.org/10.5281/zenodo.14893904 and is publicly available at https://anaconda.org/bioconda/icfree-ml as of the date of publication.
• All Galaxy wrappers have been deposited at Zenodo at https://doi.org/10.5281/zenodo.14893963 and is publicly available at https://toolshed.g2.bx.psu.edu as of the date of publication.
• The Galaxy workflow *AI-CellFree - Init* has been deposited at Zenodo at https://doi.org/10.5281/zenodo.14901023 and is publicly available at https://galaxy-synbiocad.org/u/joan/w/icfree-init as of the date of publication.
• The Galaxy workflow *AI-CellFree - Core* has been deposited at Zenodo at https://doi.org/10.5281/zenodo.14899706 and is publicly available at https://galaxy-synbiocad.org/u/joan/w/icfree-core as of the date of publication.
• The ChatGPT prompts for writing the Sampler module are publicly available at https://chat.openai.com/share/a6d8da15-3f78-434d-955e-efb7996ed07e as of the date of publication.
• The ChatGPT prompts for writing the Designer module are publicly available at https://chat.openai.com/share/56f141fe-0a97-412d-a782-39c2bf74aee6 as of the date of publication.
• The ChatGPT prompts for writing the Instructor module are publicly available at https://chat.openai.com/share/78f69117-75fb-4a51-83a7-813fd1ef0c98 as of the date of publication.
• The ChatGPT prompts for writing the data Extraction code (Learner module) are publicly available at https://chatgpt.com/share/e/66e43b41-7330-8007-9d77-50a2e1f1c9ef as of the date of publication.
• The ChatGPT prompts for writing the data Calibration code (Learner module) are publicly available at https://chatgpt.com/share/e/67152c45-22b8-8007-9f72-cc160accea8e as of the date of publication.
• Any additional information required to reanalyze the data reported in this article is available from the lead contact upon request.

## Acknowledgments

We would like to acknowledge funding provided by the 10.13039/501100001665ANR funding agency, grant numbers ANR-20-BiopNSE (ICFREE project), ANR-22-PEBB-0008 (PEPR B-BEST France 2030 program), ANR-21-ESRE-0021 (ALADIN project), and the UE HORIZON BIOS program (grant number 101070281). We also acknowledge M. Sabeti-Azad for her contribution to designing plasmids, L. Brunet for preparation of the euCFPS biological material, and Synthelis Biotech company (partner of the ICFREE project), for their contribution and the production of bacterial cell-free lysates. Finally, this work was supported by Ile-de-France DIM BioConvergence for Health.

## Author contributions

Conceptualization, J.H., J.L.F., and B.P.; methodology, B.P., A.E.S., M.M.K, A.N.H., and J.H.; software, J.H. and A.N.H.; formal analysis, A.N.H.; investigation, B.P., M.M.K., and J.L.F.; writing – original draft, J.H. and B.P.; writing – review and editing, M.M.K., J.L.F., A.N.H., A.E.S., and B.P.; funding acquisition, J.L.F.; resources, M.M.K., A.E.S., and B.P.; supervision, J.H. and J.L.F.

## Declaration of interests

The authors declare no competing interests.

## Declaration of generative AI and AI-assisted technologies in the writing process

During the preparation of this work, the author(s) used ChatGPT and DeepL to check English and generated parts of Figure 1. After using this tool/service, the author(s) reviewed and edited the content as needed and take(s) full responsibility for the content of the publication.

## STAR★Methods

### Key resources table

**Table:** 

REAGENT or RESOURCE	SOURCE	IDENTIFIER
**Antibodies**		

anti-6xHistidin-tag	Sigma	Cat#SAB2702220; RRID: AB_3095553
anti-GFP-tag	Cell signaling	Cat#2555; RRID: AB_10692764
anti-HiBiT-tag	Promega	Cat#N720A

**Bacterial and virus strains**		

Escherichia coli DH5*α*	Lab stock	
Escherichia coli BL21 (DE3)	Lab stock	
Escherichia coli W3110	Lab stock	

**Biological samples**		

E. coli cell-free lysates	Synthelis Biotech	
HeLa Lysate	ThermoScientific	Cat#88882

**Chemicals, peptides, and recombinant proteins**		

HEPES	Sigma-Aldrich	Cat#H3375
Nucleotide mix - Adenosine 5-triphosphate dipotassium salt hydrate	Sigma-Aldrich	Cat#A8937
Nucleotide mix - Guanosine 5ʹ-Triphosphate, Disodium Salt	Sigma-Aldrich	Cat#371701
Nucleotide mix - Sel disodique cytidine-5′-triphosphate, 98+ %	Thermo Scientific Chemicals	Cat#J62238.ME
Nucleotide mix - Uridine 5′-triphosphate, sel trisodique, hydraté, 90%	Thermo Scientific Chemicals	Cat#226310010
3- PGA (Acide D-(−)-3-phosphoglycérique disodium salt)	Sigma-Aldrich	Cat#P8877
Spermidine	Sigma-Aldrich	Cat#85558
Creatine phosphate crystalline, sup à 97%	Sigma-Aldrich	Cat#10621714001
PEG 8000	Sigma-Aldrich	Cat#89510
Mg-glutamate	Sigma-Aldrich	Cat#49605
K-glutamate	Alfa Aesar	Cat#A17232
Amino acids - L-Alanine	Sigma	Cat#A7627
Amino acids - L-Arginine	Sigma	Cat#A5006
Amino acids - Glycine	Sigma	Cat#G7126
Amino acids - L-Histidine	Merck	Cat#1.04351.0100
Amino acids - L-Lysine	Sigma	Cat#L5626
Amino acids - L-Proline	Sigma	Cat#P0380
Amino acids - L-Serine	Sigma	Cat#S4500
Amino acids - L-Threonine	Sigma	Cat#T8625
Amino acids - L-Valine	Sigma	Cat#V0500
Amino acids - L-Aspartic Acid	Sigma	Cat#A9256
Amino acids - L-Asparagine anhydrous	Sigma	Cat#A0884
Amino acids - L-Cystine	Sigma	Cat#C8755
Amino acids - L-Glutamine	Sigma	Cat#G3126
Amino acids - L-Leucine	Sigma	Cat#L8000
Amino acids - L-Methionine	Sigma	Cat#64319
Amino acids - L-Tryptophan	Sigma	Cat#T8941
Amino acids - L-Tyrosine	Sigma	Cat#T3754
Amino acids - L-Phenylalanine	Sigma	Cat#78019
Amino acids - lsoleucine	Sigma	Cat#I7403
BsmBI-v2	NEB	Cat#R0739S
BbsI	NEB	Cat#R0539S
BsaI	NEB	Cat#R3733S
DpnI	NEB	Cat#R0176L
BamHI	Promega	Cat#R6021
EcoRV	Promega	Cat#R6351
T4 DNA ligase	Promega	Cat#M1801
T4 DNA ligase	NEB	Cat#M0202
Ampicillin	Sigma–Aldrich	Cat#A1593
(IPTG) isopropyl -*d*-1-thiogalactopyranoside	Sigma–Aldrich	Cat#I5502

**Critical commercial assays**		

Q5 High-Fidelity 2X Master Mix	NEB	Cat#M0492L
PCR purification kit	NEB	Cat#T1130
NucleoBond Xtra Maxi kit	Macherey-Nagel	Cat#740414.1
Nano-Glo® HiBiT Lytic Detection System	Promega	Cat#N3030
Nano-Glo® HiBiT Lytic Detection System	Promega	Cat#N3040

**Deposited data**		

icfree-ml source code	https://github.com/brsynth/icfree-ml	https://doi.org/10.5281/zenodo.14900104
icfree-ml conda packages	https://anaconda.org/bioconda/icfree-ml	https://doi.org/10.5281/zenodo.14893904
icfree-ml Galaxy wrappers	https://toolshed.g2.bx.psu.edu	https://doi.org/10.5281/zenodo.14893963
*AI-CellFree - Init* Galaxy workflow	https://galaxy-synbiocad.org/u/joan/w/icfree-init	https://doi.org/10.5281/zenodo.14901023
*AI-CellFree - Core* Galaxy workflow	https://galaxy-synbiocad.org/u/joan/w/icfree-core	https://doi.org/10.5281/zenodo.14899706

**Experimental models: Organisms/strains**		

Escherichia coli DH5*α*		
Escherichia coli BL21 (DE3)		
Escherichia coli W3110		

**Oligonucleotides**		

See Table S3		

**Recombinant DNA**		

Colicin M	Synthesized by GeneCust	
Colicin E1	Synthesized by GeneCust	
GFP1-10	Synthesized by GeneCust	
GFP11	Synthesized by GeneCust	
His-GFP1-10	This study	
His-GFP11-Colicin M	This study	
His-GFP11-Colicin E1	This study	
His-HiBiT-Colicin M	This study	
His-HiBiT-Colicin E1	This study	
His-HiBiT-sfGFP	This study	

**Software and algorithms**		

Echo Cherry Pick software	Beckman Coulter	
Protocol Designer	Opentrons	
Image Lab™ Software	Bio-Rad Laboratories	
ChatGPT-4	Openai	

### Experimental model and study participant details

*Escherichia coli DH5α* has been used in the following conditions: LB broth supplemented with 100 μg/mL of ampicillin for maintaining the plasmids and grown at 37°C with shaking (180 rpm).

*Escherichia coli BL21 (DE3)* has been used in the following conditions: LB broth supplemented with 100 μg/mL of ampicillin for maintaining the plasmids and grown at 37°C with shaking (180 rpm).

*Escherichia coli W3110* has been used in the following conditions: LB and grown at 37°C with shaking (180 rpm).

### Method details

#### Generating ready-to-use code by ChatGPT

To create the different mixes, we needed to transfer volumes of solution from a source well-plate to a destination well-plate. The source plate is filled by hand while the destination plate is filled with the liquid handler ECHO 650. Because this device can handle volumes multiple of 2.5 nL, we consider only such values for volumes to test. Therefore, discrete ranges must be considered as solutions space for each component, making it a finite set of solutions while remaining very large (e.g., millions of possible solutions for a few components). We can now generate a set of values to test within the component’s discrete ranges. Since we must handle duplicates or triplicates, generating 100 samples to test at each AL loop seems to be a good size. As such a subset is very small compared to millions of possible values, random values are not representative of the solution space since two random values can be close to each other. Therefore, because the quality of the first tested values can have a dramatic impact on the efficiency of AL, we preferred to use LHS that provides a representative sampling of the full solution space.^41^ Then, this module takes as input a file containing the list of components in the cell-free system with their possible maximum volumes and generates a set of LHS-based samples. To write this code, we use ChatGPT-4 (Data Analyst customized GPT) by feeding it with the input file (Data S1) and non-technical language was used for prompts. After few iterations, the code generated by ChatGPT-4 was executed to get the sampling file with the following settings: number of samples was 100 for proCFPS and 50 for euCFPS. For colicin M in proCFPS, ratios of 0, 0.2, 0.4, 0.6, 0.8, and 1 were used, whereas fixed steps of 20 were applied for colicin E1 in proCFPS and colicin M in euCFPS. Control and calibration buffers are added to the output sampling file.

The conversion into ECHO instructions can be done from other data than a sampling of volumes. One would like to generate instructions from source and destination plate designed by hand or provided from another process. Then, we decided to implement the plate Designer module that transforms sampling of volumes into source and destination plate. In the same way, we used ChatGPT-4 to code this module by giving the sample volumes file (Data S2) and non-technical language was used for prompts. After some iterations, the code generated by ChatGPT-4 was executed to get the source and destination files with plate dimensions of 16 x 24, number of replicates set at 4, 5, or 6. The dead volume was 20,000 nL for the source wells and sample volumes were at 6,500 nL for proCFPS and 2,000 nL for euCFPS, where the full reaction was dispensed by ECHO.

The Instructor module takes source and destination plates as inputs and writes ECHO instructions. Similarly, ChatGPT-4 has been used to write the code by passing the source and destination files (Datas S3 and S4) and non-technical language was used for prompts. After few iterations, the code generated by ChatGPT-4 was executed to get the instructions file (Data S5) with a maximum transfer volume of 1,000 nL and split threshold of 1,000 nL for all the CFPS systems used. Efficient transfers were ensured by adjusting the ECHO transfer mode for the viscous and critical components by setting *384 PP_AQ_CP* as source plate type.

At each AL loop, the Experimenter module was carried out at the wet lab and has provided luminescence or fluorescence measurements. Then, within the Learner module, fluorescence values were extracted from plate reader’s output file (Data S6), and due to cell-free variability, a calibration process was needed to standardize fluorescence levels of one plate in relation to the others. This step was completed with two pieces of codes written by ChatGPT-4 with a non-technical language for prompts. After iterating, the codes generated by ChatGPT-4 for extracting and calibrating were executed to calibrate fluorescence data from one plate to another (Data S7). All these data are presented respectively for luminescent-based assays for euCFPS (Datas S8–S14). The calibration algorithm is detailed in Document S1.

#### Experimental preparation

For prokaryotic gene expression, *ColM* and *ColE1* sequences (coding sequenses in Table S2) were synthesized by GeneCust in pUC57 vectors. These were subsequently cloned into *pIVEX* vectors, the fully annotated plasmid sequences are available in materials availability section. PCR amplification of the colicin sequences and *pIVEX* backbone was performed using the primers provided in the Table S3 and then digested by BsmBI and BbsI for a golden gate assembly. Similarly, the *GFP1-10* and *His-GFP11* fragments were cloned into *pIVEX* vectors, following the same procedure as for the colicins. Then, *His-GFP11* was used as a tagging system (Figure S7) by using BbsI to digest the *pIVEX* vector containing *His-GFP11*, and BsaI was used for the colicin sequences. All restriction enzymes used were sourced from NEB. After each digestion, a DpnI treatment (NEB #R0176L) was performed for 1 h to degrade any residual template DNA, followed by purification using the NEB PCR purification kit (NEB #T1130). The purified fragments were then ligated using T4 DNA ligase (NEB #M0202) according to the manufacturer’s protocol. Following ligation, the recombinant plasmids were transformed into chemically competent *E. coli DH5α* cells. Concerning eukaryotic gene expression, the *p0-EMCV-renilla* coding for Renilla luciferase was described previously.^13^ The *p0-EMCV-His-HiBiT-GFP*, *p0-EMCV- His-HiBiT-ColM* and *p0-EMCV- His-HiBiT-ColE1* constructs were cloned inserting the *His-HiBiT-GFP*, *His-HiBiT-ColM* and *His-HiBiT-ColE1* fragment previously digested by BamHI (Promega, #R6021) and EcoRV (Promega, #R6351) restriction enzymes from bacterial *pET21* counterparts. Fragments were inserted into p0-EMCV with T4 DNA ligase (Promega, #M1801). This mix was incubated for 1 h at room temperature or one night at 16°C. After ligation, bacterial transformation was done for each ligation mix. The recombinant plasmid was selected with Luria broth (LB) agar with ampicillin after one night at 37°C. The plasmids *GFP1-10*, *His-GFP11-ColM*, *His-GFP11-ColE1* for the prokaryotic system, in addition to the plasmid *p0-EMCV- His-HiBiT-ColM* for the eukaryotic system were prepared at enough amount for all rounds of experiments. The plasmids were isolated from a 300 mL LB of *E. coli DH5α* using the Plasmid DNA purification NucleoBond Xtra Maxi of Macherey-Nagel. The plasmid pellets were resuspended in 1 mL nuclease and free water and stored at −20 C. They were pooled to reach a concentration of ∼50 nM to be able to be transferred by ECHO and then aliquoted for all the round/loop experiments avoiding any variation due to different batches.

The *pET 15b-6His-HiBiT-GFP* plasmids were used to transform the *E. coli BL21 (DE3)* competent cells by a heat shock method (42°C). Then, (LB) medium was added, and the transformed cells were grown at 37°C for 1 h. After overnight incubation at 37°C with shaking (180 rpm), transformants cells were selected on the LB agar medium supplemented with ampicillin^42^ (100 μg/mL) (Sigma–Aldrich). To produce recombinant His-HiBiT-GFP, *E. coli BL21 (DE3)* precultures, harboring plasmids mentioned above, were used to inoculate 1% (v/v) of LB broth supplemented with 100 μg/mL of ampicillin and grown at 37°C with shaking (180 rpm), until reaching an OD600 of 0.6–0.8. Expression was induced by addition of 1 mM isopropyl -*d*-1-thiogalactopyranoside (IPTG). Then *E. coli BL21-pET 15b-6His-HiBiT-GFP* cells were incubated for three additional hours at 37°C with shaking at 180 rpm. After expression, cells were collected by centrifugation (3,000 rpm, 20 min at 4°C) and re-suspended in lysis buffer (100 mM TRIS-HCl pH 7.5, 500 mM NaCl and 10% glycerol). Finally, cells were lysed by sonication on ice (4 × 20 s pulse on, 20 s pulse off; 70% amplitude) and cell debris were removed by centrifugation (20,000 rpm, 20 min at 4°C). After the lysis step, the lysates were loaded onto a column containing Nickel resin grafted on a nitrilo-tri-acetic (Ni-NTA) matrix (Cytiva) previously equilibrated with lysis buffer. Then, they were washed by lysis buffer supplemented with 25 mM imidazole and eluted by a linear gradient from 25 mM to 500 mM imidazole. To remove the imidazole, a desalting step was performed using PD miditrap columns (Cytiva) as recommended by the supplier. The purity of each recombinant protein was checked on SDS-PAGE and the concentration was determined by Bradford method (Bio-Rad).

#### Routine experimental setup (experimenter module)

The experimental design was based on the composition of the reaction mixture that was independent considering proCFPS from euCFPS. ProCFPS comprised essential components including Mg- and K-glutamate, amino acids, spermidine, 3-phosphoglyceric acid (3-PGA) or creatine phosphate (CP) as the energy source, nucleoside triphosphates (NTPs), PEG-8000, HEPES, two plasmid DNA constructs (GFP1-10 and GFP11-ColM or GFP11-ColE1) for co-expression, and a commercial cell extract (Syn-Xtract Cell-free lysates) by Synthelis Biotech company. Certain components, such as tRNA, coenzyme A (CoA), nicotinamide adenine dinucleotide (NAD), cyclic adenosine monophosphate (cAMP), and folinic acid, which have been shown to have a minimal impact on protein production, were excluded from the reaction mixture.^24^ EuCFPS was a commercial system consisting of four components namely HeLa based lysate, accessory proteins, reaction mix, and our plasmid DNA construct with HiBiT fused to colicin M (HiBiT-ColM).

The two key tools for high-throughput and low-volume platform installation are the technological breakthrough leads by the ECHO acoustic liquid handler, that can distribute repetitive steps of 2.5 nL liquid droplets altogether with a compatible microplate reader (Figure S2A). As a multiple of 2.5 nL, we suggested that to dispense volumes between 20 nL and 1000 nL by the ECHO liquid handler, the 1000 nL was our threshold use above which we split the dispensing if we need more than 1000 nL of the same component in the same well (Figures S2B and S2C). Bigger fixed volumes above 2 μL were distributed by electronic multichannel pipette (Eppendorf Xplorer plus). A microplate centrifuge associated with the platform increases the reliability of microplate reactions. As most of assembled reactions and specifically CFPS reactions need incubation at a controlled temperature for a better reproducibility of results, the incubator of the Synergy HTX reader was used for incubating the assembled destination plate after being sealed with the transparent adhesive sheet (Sigma–Aldrich #A5596). The manual multichannel pipette injection into the 384-well destination plate takes less than 5 min, 4 μL of cell-free lysate and remaining water per proCFPS reaction and 2 μL luciferase substrate for each euCFPS reaction. The ECHO assembly of the 10 reagents in proCFPS (including water) with their variable volumes to reach a final volume of 6.5 μL for the 384 wells took around 3.5 h, while transferring the 5 reagents (including water) for euCFPS by ECHO did not exceed 45 min. These delays are perfectly suitable to investigate concomitantly hundreds of combinations of reagents assembly and feed AL loop.

The experiments using the proCFPS aimed to optimize the production of colicin M and E1, and this was achieved by varying the concentrations of the TX-TL buffer components. The cell-free components were prepared as one batch from each component that is enough for all the experimental iterations and aliquoted with stock concentrations optimized to be transferred by the ECHO 650 liquid handling system. The commercial bacterial cell-free lysate was provided by Synthelis Biotech company, and the reactions were performed in 10.5 μL volumes at 30°C in a 384-well plate. Mg- and K-glutamate were individually standardized through preliminary experiments for the two different batches of cell lysate used for either colicin M or Colicin E1 system (Figure S8). All the cell-free reaction components were set to be at different concentrations/ratios except the lysate at 33% of the reaction, and HEPES at 50 mM as the buffering reagent. 3-PGA was used as an energy source for the GFP-Colicin M system, while creatine phosphate was used for the GFP-Colicin E1 system with 100 mM as a final reference concentration^43^ after testing other alternatives to 3-PGA as the energy source using the GFP-Colicin M plasmids (Figure S9). It was critical to ensure that the maximum volumes from all components together did not exceed the total reaction volume (10.5 μL) and that each component was not below 20 nL. These components underwent a drop test to determine the correct transfer mode calibration to ensure that only uniform droplets are at the center of the destination wells. The 384-well polypropylene (PP) microplate (PP-0200) was chosen as the source plate, while the calibration transfer mode was ‘384 PP_AQ_GP3’ for all reagents except for K-glutamate, creatine phosphate, NTPs, and DNAs for their viscosity, and instead using the ‘384 PP_AQ_CP’ calibration mode. Cell lysate was added to the 384-well destination plate (Nunc 384-well optical bottom plates polystyrene (PS), Thermo-Scientific #242764) using a multichannel pipette (Eppendorf Xplorer plus #4861000778) after the transfer of all the other components by ECHO. We used the standard concentrations^40^ as our 100% concentration level for all TX-TL components, which served as the reference buffer composition, giving a yield value of 1. For the colicin M system, the maximum final concentrations for the two plasmid DNAs (*GFP1-10* and *GFP11-ColM*) were 6 nM, while for the colicin E1 system, the concentrations of *GFP1-10* and *GFP11-ColE1* were 5 nM.

After generating the instructions file, the components were added manually in their positions in the source plate based on the source plate map file. The ECHO’s ‘’Cherry Pick’’ software is used for selecting the appropriate source and destination wells by uploading the instructions file to initiate the transfers. During this process, dispensing errors may occur and are represented as an “Exceptions sheet” by the ECHO, that must be promptly resolved using Excel macros to ensure the successful transfer of all reagents in the specified volumes. Then, the coupled TX-TL reaction was left incubated for 24 h at 30°C in the Synergy HTX BioTek reader. The fluorescence kinetics were measured with the excitation wavelength fixed at 485/20 nM, and the emission at 528/20 nM, with the gain set at 50 for colicin M and at 35 for colicin E1, with continuous linear shaking at slow speed of 360 cpm. The fluorescence was measured from the bottom of the 384-well plates sealed with transparent sealers, and only the readings of the 24h endpoint were used to analyze and process the data further.

The commercial HeLa CFPS kit (Thermo scientific, #88882) was used for the optimization loops. To reduce the costs and save expensive reagents, we aimed to lower the cell-free reaction volume to 2 μL which allowed us to use one fresh kit per loop. We ordered enough kits in one batch and verified that all the components across each kit had the same Lot Number, ensuring that we work with the same aliquots for each round to eliminate variation across the rounds. Following the manufacturer instruction ratios for the different components, the euCFPS assembly was done with 1 μL of HeLa lysate, 0.2 μL of accessory proteins, 0.4 μL of reaction mix, 0.2 μL of cloned DNA and 0.2 μL of water in a total reaction volume of 2 μL to make the final input mass of DNA 80 ng in our reaction volume. Each of these components underwent a drop test on ECHO to determine the best calibration mode that transfers uniform droplets in the center of the destination wells. The calibration transfer mode was 384 PP_AQ_GP3 for all reagents except for HeLa Lysate using 384 PP_AQ_CP. However, we faced some dispensing limitations by ECHO with the high stock concentrations of some reagents; as the *HiBiT-ColM* plasmid DNA in this case, which we need its final concentration to be 14.2 nM for the 2 μL reaction to correspond with the recommended input mass by the kit. By performing trial and error drop transfer tests by the ECHO, we obtained the best results of uniform droplets from a maximum stock concentration of 55 nM of the DNA transferred from the source plate, while higher stock concentrations were not transferred at all by ECHO. Consequently, the recommended volumes by the kit (corresponding to 1X concentration) were not possible to be achieved, as the volume of DNA needed would be larger to fit the required 2 μL. Thus, we made a preliminary test to assess the performance of half the recommended concentrations by the kit as 0.5X, till the maximum concentrations possible in 2 μL as 0.925X. The reaction was achieved with the 0.5X volumes making this as our new reference point for the euCFPS AL loops (Figure S10).

The initial round (Loop 0) for euCFPS was done with 56 tests (including 6 control tests) in 4 replicates, giving 224 wells in total. We chose to limit the number of wells due to the evaporation issues that occurred in preliminary tests during the dispensing time of the exceptionally low volumes like 2 μL. As the recommended incubation time by the kit for the transcription coupled translation reaction is from 1.5 to 6 h, we incubated the reaction in a sealed plate in the reader for 4 h at 30°C starting with 1 min orbital moderate speed shaking at 207 cpm followed by two intermittent shaking steps for 30 s at the same speed every 2 h. Then, the plate was centrifuged, and the adhesive sheet was removed. HiBiT activity was measured using the Nano-Glo HiBiT Lytic Detection System (Promega #3030), we used a new kit only dedicated for all the euCFPS tests and rounds. This activity was measured by adding equal volume of the HiBiT Lytic Reagent mixture of 2 μL with the 16-channel pipette, then incubated for 10 min (including 2 min of orbital shaking as a first step at 207 cpm) in the Synergy HTX reader at 30°C and measured with 1 s of integration time without measuring interval at gain 150 from top with 4.5 mm reading height.

#### Routine active learning (experimenter module)

In Experimenter module, a surrogate model was trained using Gaussian Process (GP) regression, using input as the volumes of the cell-free mix components (presented in Table S1); and output as calculated yield or directly measurements of fluorescence or luminescence. The GP model was chosen due to its ability to perform effectively on small datasets,^44^ and our proCFPS and euCFPS systems have 11 and 4 components, respectively (Figure S11). Moreover, it provides reliable uncertainty estimates, which are crucial for exploration in AL frameworks. While ensemble-based multilayer perceptron (MLP) models and XGBoost are powerful machine learning approaches widely used in other studies, they are more susceptible to overfitting when trained on limited data. Their performance typically improves with larger datasets, making them less suitable for early-stage AL applications. Then, the model has been validated to ensure it accurately predicts system performance based on the components' values (Figure S12). In this paper, we utilized Expected Improvement^45^ (EI) as an AL acquisition function to select the top 30 or 50 most informative samples from a much bigger pool of 1000 or more randomly generated samples within a specified search space (Figure S13). This method is consistent with the Bayesian optimization framework for regression,^44^ aiming to enhance model performance by identifying the best target and highest uncertainty, rather than using the margin as a metric for classification tasks.^32^ The m data points are ranked based on their EI value, with the highest values indicating the most informative samples for the model. After the n new points are labeled after lab experiments, they are added into the training set, and we retrain our GP regressor for the next loop. Also, two training methods have been compared in this study: vanilla active learning (VAL) and CM methods. VAL selects the top 30 or 50 points from the search space based solely on their acquisition EI value, without considering the diversity of the data (Figure S14). In contrast, the CM method selects only 15 or 20 points, focusing on both informativeness and diversity. As training progressed, the Pearson correlation value increased significantly for both methods, indicating that each was successful in selecting informative data points for training. Notably, while the CM method selected 50 to 60% fewer data points than VAL, it achieved similar model performance for the colicin M and E1 in ProCFPS system, or even higher performance in the colicin M in euCFPS system (Figure S15). Indeed, by comparing the average yield across multiple repetitions of each experiment, we observe that there is no statistically significant difference in the highest yield obtained between VAL and CM. This result indicates that CM is also capable of identifying the highest yield, even with fewer data points or fewer experimental iterations (Figure S16). The relationship between yield and individual components, along with their importance in model predictions is further detailed in Figure S17. This demonstrates that increasing the amount of training data is not always necessary for model improvement. The traditional EI function is useful for evaluating the informativeness of individual data points but is less effective when applied to batch queries, where diversity plays a crucial role. The CM method overcomes this limitation by ensuring that selected data points are not overly like one another, resulting in a more diverse and representative dataset for training. This not only enhances model efficiency but also reduces resource consumption by minimizing the inclusion of low-performing samples. After four loops, the process was concluded, as further improvements in yield were not observed. However, it remains possible that additional iterations could increase the model’s accuracy. To enhance data representation through selected samples, we used the Cluster Margin method on top of the EI acquisition function. Initially, the entire dataset is segmented into k groups (Figure S18) using Hierarchical Agglomerative Clustering (HAC), where samples within each group are considered sufficiently similar. This clustering method utilizes Average Linkage to measure distances between points, specifically avoiding the chaining effect associated with Single Linkage Clustering.^46^ Single linkage clustering can force clusters together due to single elements being close, despite many other elements within each cluster being distant from each other. We selected EI as a measurement like in VAL, but following the selection of these examples, the clusters are utilized to pick the most diverse set of n points (Figure S18) using a round-robin sampling strategy. These points are separated into their groups from HAC, and these clusters are then ordered by size, starting from the smallest to the largest. From the first cluster, the point with the highest EI is selected and repeated to the next bigger cluster until n points are chosen. This strategy ensures that the selected points are not only uncertain (as indicated by high EI) but also representative of different clusters or regions within the dataset.

Then, the Active Learning loop was run following the following steps.

(1) *Sampling*: Exploring the design space by iteratively selecting top few new points using the EI acquisition function to measure and rank the value of each new point to add into the model;
(2) *New Experimentation*: Conducting new experiments based on the selected points;
(3) *Data Aggregation*: Adding the results of the new experiments to the existing dataset;
(4) *Model Update*: Retraining of the surrogate model with the updated dataset;
(5) *Convergence Check*: Assessing if the model performance or optimization goal meets a predefined convergence criterion (performance plateau in this work).

To define our search space, we took the maximum volumes^40^ for each component of the cell-free system as shown in Table S1, these volumes were used in the input parameters or components file, then we initialized a design space based on the ranges of each component and selected an initial set of 100 experiments to run by using the Sampler module with the following command: *python -m icfree.sampler component_maxValues.tsv sampling.csv 100 --seed 42 --fixed_values ‘{“HEPES”: 10}’ --ratios “0,0.2,0.4,0.6,0.8,1”*, where: *component_maxValues.tsv* is the name of the file containing the maximum volumes of the cell-free components; *sampling.csv* is the name of the output file; *100* is the number of samples generated; *42* is the seed number for reproducibility purpose; *‘{“HEPES”: 10}’* is to fix the value of HEPES component to 10 mL; “*0,0.2,0.4,0.6,0.8,1”* is the list of the ratios to take as possible volume values for each of the components. When the program ended, we obtained the 100 first cell-free combinations and we conducted this initial set of experiments to get the starting dataset. This was performed similarly for the euCFPS but without fixed volume values for any of the components, and the experiments were initiated using 50 samples.

### Quantification and statistical analysis

In Figure 2D, data are presented as means ± SD for six independent experiments (*n* = 6) using a unique batch of recombinant his-HiBit-GFP purified proteins.

In Figure 3, each dot in swarm plots (A), (C), and (E) presents the mean of one experiment through 3 repetitions, while the red line is average of all experiments within one loop. Regarding graphs (B), (D), and (F), each column with error bar presents mean ± SD for 3 repetitions of the same experiment.

### Quantification and functionality assessment

A range of recombinant His-HiBiT-GFP protein has been produced of 0 at 10 ng of protein. Each point of the range of recombinant protein was denatured in the Laemmli buffer at 75°C for 10 min, and resolved on a 10% Sodium Dodecyl Sulfate Polyacrylamide gel (SDS-PAGE) and transferred to a Polyvinylidene fluoride (PVDF) membrane. Membranes were blotted using anti-6xHistidin-tag (Sigma #SAB2702220), anti-GFP-tag (Cell signaling #2555) and anti-HiBiT-tag (Promega #N720A). For each blot, the Precision Plus Protein Dual Color Standard (Bio-rad) was used as a molecular ladder. Western blot image acquisition was performed using the Bio-Rad ChemiDoc MP Imaging System, using colorimetric modes for revealing molecular ladder and chemiluminescence for revealing antibodies. Colorimetric and chemiluminescence were merged using the Bio-Rad Image Lab software. Fluorescence activity was measured under 485 nm excitation wavelength and 535 nm emission wavelength. The gain was automatically calculated from the highest recombinant GFP protein concentration with 30 flashes and 40 μs of integration time at a temperature of 30°C. HiBiT activity was measured using the Nano-Glo HiBiT Lytic Detection System (Promega #3040). This activity was measured with 2 μL of translation mixture after 10 min of incubation with 2 μL HiBiT substrate and measured with 50 ms of integration without measuring interval. Both luminescence and fluorescence were read under a TECAN microplate reader (spark model, Switzerland) using p384 microplate black and low volume (Greiner #784900).

The evaluation of the antimicrobial activities of the synthesized peptides employed a systematic and automated approach. ECHO and Opentrons OT-II liquid handler, equipped with a HEPA filter, facilitated precise liquid handling in a high-throughput manner. Susceptible *E. coli W3110* cells (50 μL) were thoroughly mixed using Opentrons, incorporating varying volumes of cell-free reaction mixtures containing the antimicrobial proteins dispensed by ECHO into a 384-well plate. ProCFPS reactions (1, 5, 10 μL) and euCFPS counterparts (1, 2, 4, 8 μL) were introduced accordingly. Setting a baseline, an optical density (OD600) of 0.001, achieved by diluting overnight bacterial cultures, and a blank LB media control were incorporated. The automated procedure involved the initial mixing of cells, followed by the transfer of the cell mixture to designated wells at a default flow rate of 94 μL/s. To enhance reliability and minimize bubble formation, dispensing and subsequent mixing of cells with the cell-free reaction mixture were repeated twice at a reduced flow rate of 5 μL/s. Incubation of cells was done for 24 h at 37°C with continuous orbital shaking at a fast speed.
