# Supplementary material for: An AI-driven workflow for the accelerated optimization of cell-free protein synthesis
Source: iScience. 2025 Sep 19;28(10):113599. doi: 10.1016/j.isci.2025.113599 (PMC12529354; doi:10.1016/j.isci.2025.113599)
Supplement: Document S1. Figures S1–S17 and Tables S1–S4 [file mmc1.pdf]

iScience, Volume *28*

Supplemental information

An AI-driven workflow for the accelerated optimization of cell-free protein synthesis

Mostafa M. Khalil, Aisha Elsawah, An N. Hoang, Jean-Loup Faulon, Baptiste Panthu, and Joan Hérisson

Supplemental Information

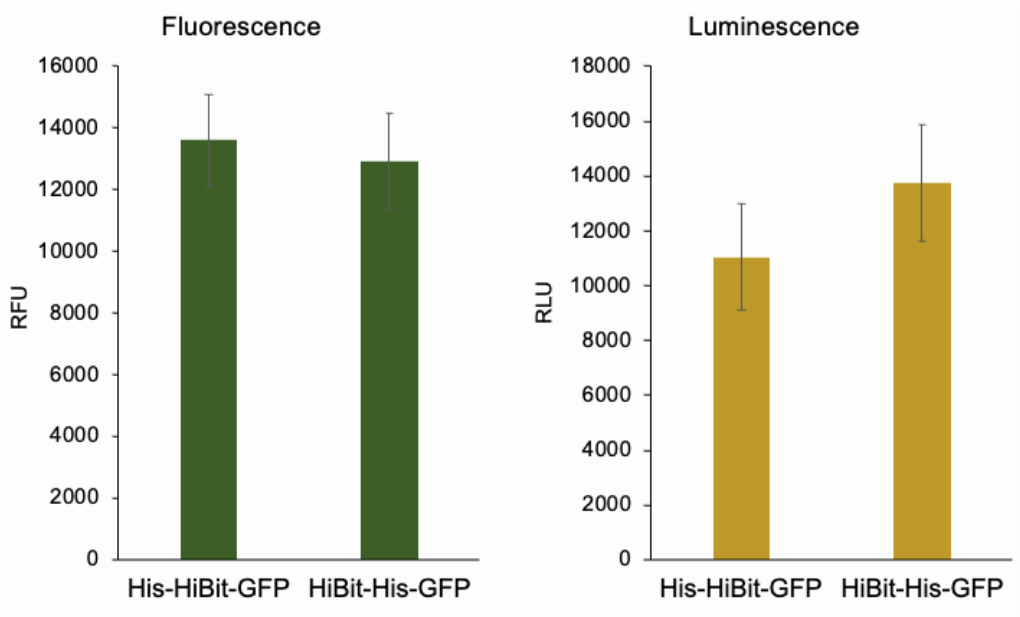

Figure S1. Effect of the N-terminal tag position on the fluorescence and luminescence of the GFP protein. Showing the fluorescence intensity (left panel in relative fluorescence units, RFU) and luminescence activity (right panel in relative luminescence units, RLU) of the two N-terminally double-tagged GFP variants: His-HiBiT-GFP and HiBiT-His-GFP. The fluorescence reflects overall protein expression levels, while luminescence corresponds to HiBiT tag complementation activity. Both measured after expression in a transcription-translation-coupled prokaryotic cell-free protein synthesis (proCFPS) system. Error bars represent standard deviation from 5 replicates experiments (n = 5).

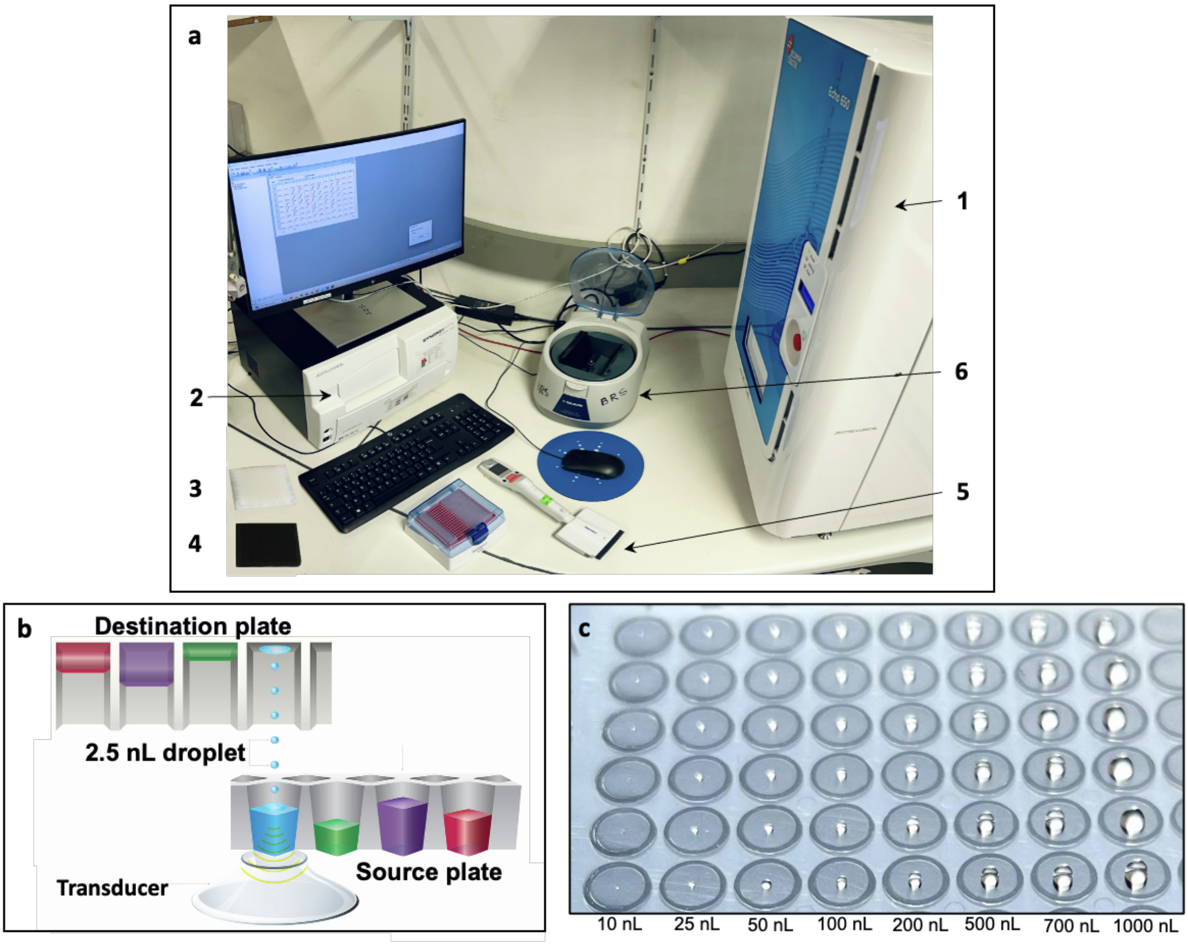

Figure S2: High-throughput and low-volume platform installation. a) The platform is assembled with an ECHO® 650 acoustic liquid handler (1) and a Synergy HTX BioTek microplate reader (2) for reaction incubation and fluorescence or luminescence measurement. A 384-well source plate (3) and a 384-well destination plate compatible with luminescence or fluorescence detection (4) are used for liquid distribution and assembly, respectively. Volumes greater than 2 µL are dispensed using an electronic 16-channel micropipette (5). A microplate centrifuge (6) enhances reaction reliability, and a plate sealer prevents evaporation. b) Principle of reagent transfer between source plate and destination plate using ECHO®. The transducer generates acoustic waves for transferring successive 2.5 nL droplets. c) Drop tests showing 48 water nanodroplets from 10 nL to 1000 nL assembled in less than 5 seconds. Testing each of the components is crucial for the reproducibility before the reaction assembly by the platform.

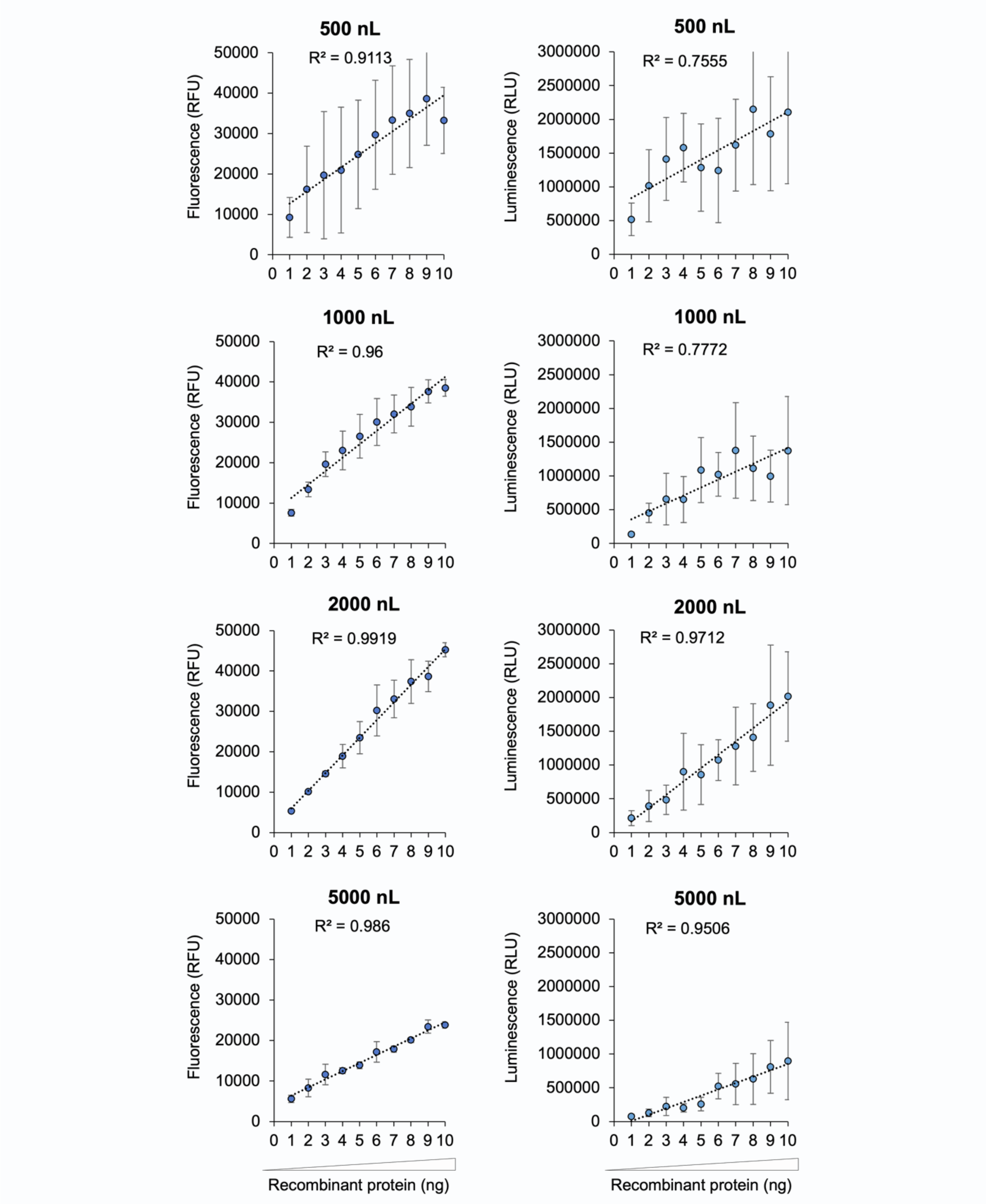

Figure S3. Sensitivity and miniaturization assessments of fluorescence and luminescence activities of His-HiBiT-GFP. Fluorescence (left panels) and luminescence (right panels) activities measured from 1 ng to 10 ng of purified recombinant His-HiBiT-GFP protein in total reaction volumes ranging from 500 to 5000 nL. Data were obtained by automation assembly using 6 replicates from the same purified recombinant protein. This setup demonstrates the feasibility of using miniaturized CFPS reactions for high-throughput screening, allowing reliable detection of low protein amounts while significantly reducing reagent amounts and experimental costs.

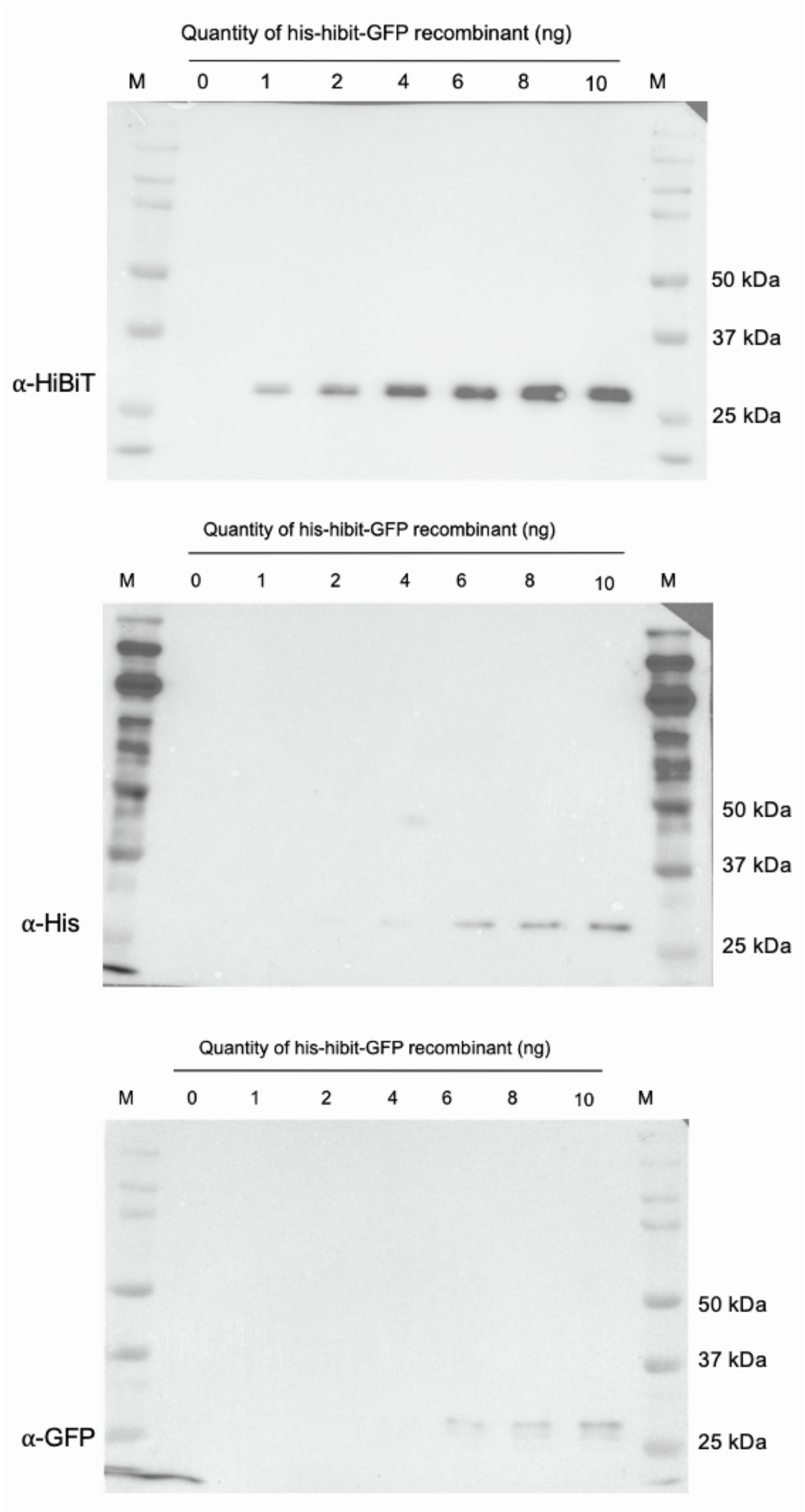

Figure S4: Uncropped gel used for figure 2d.

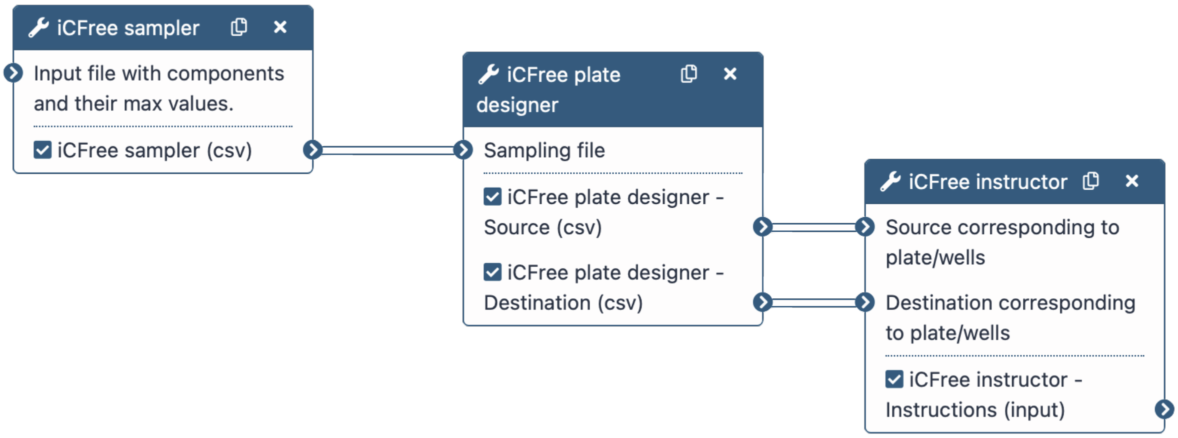

Figure S5: *AI-CellFree – Init* Galaxy workflow. This workflow, designed with the Galaxy Workflow Editor, takes as input cell-free components maximum volumes and provides ECHO® liquid handler instructions file.

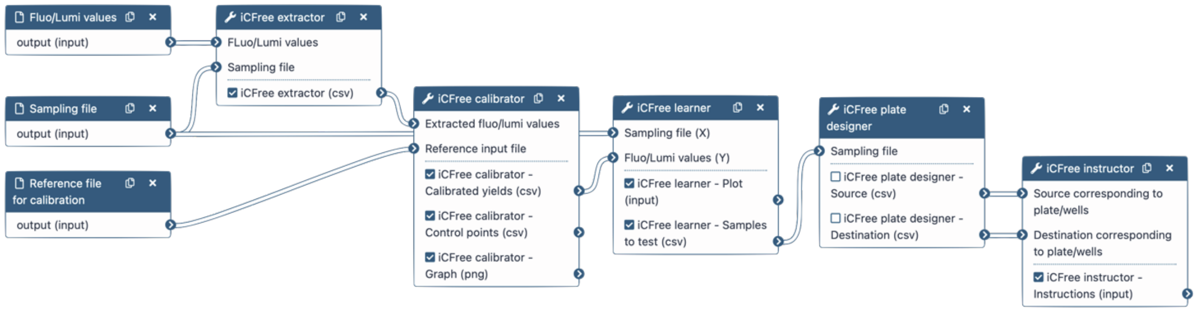

Figure S6: *AI-CellFree – Core* Galaxy workflow. This workflow, designed with the Galaxy Workflow Editor, takes as input fluorescence/luminescence values, cell-free components combinations and reference yields file for calibration, and provides ECHO® liquid handler instructions file.

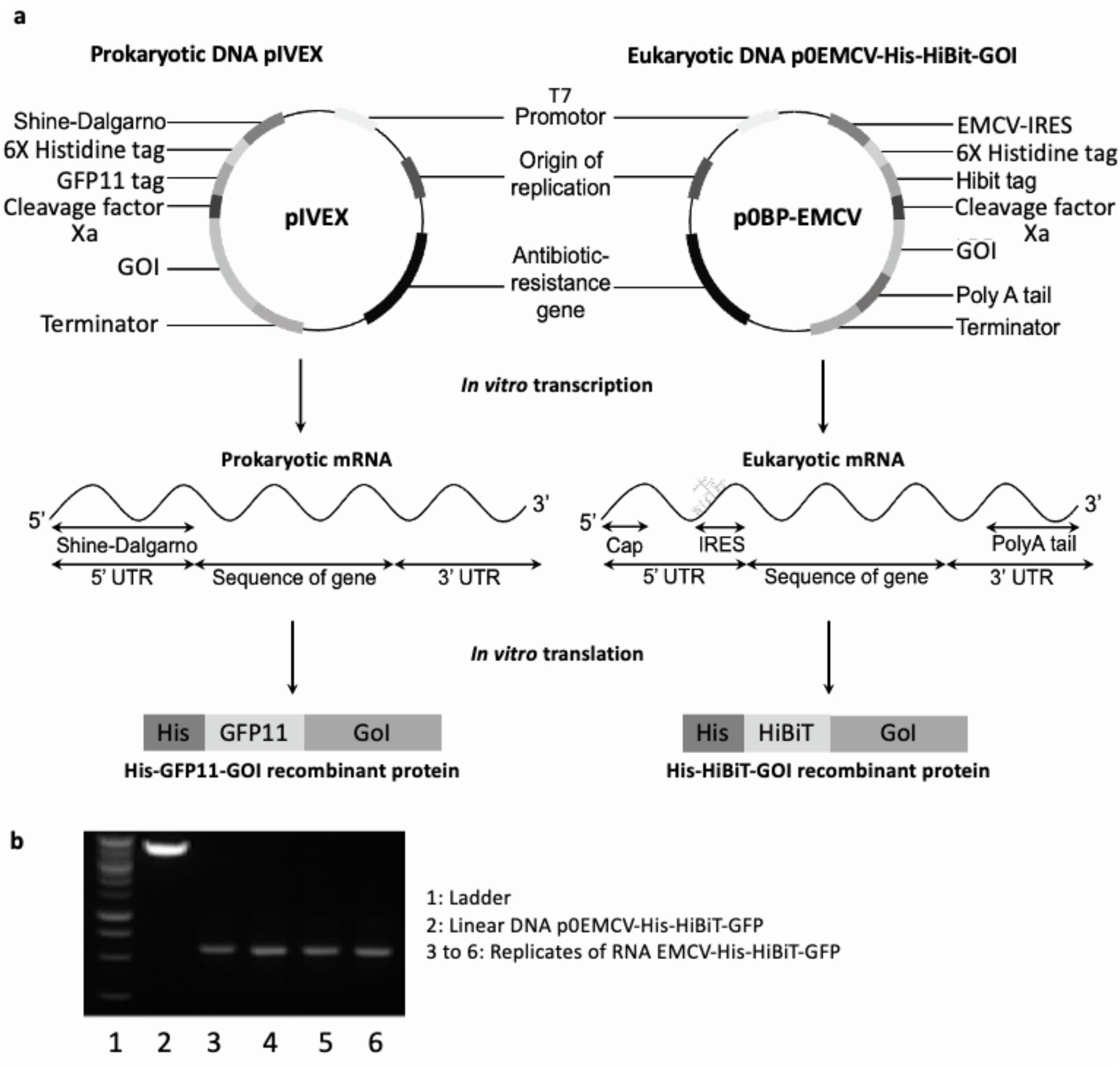

Figure S7: Split-GFP and HiBiT plasmid constructions for cell-free AMP synthesis. a) Design of two plasmids pIVEX and p0BP-EMCV for the in vitro expression of our mRNAs suitable for prokaryotic or eukaryotic translation, respectively. b) Migration of linear DNA and RNA, produced after in vitro transcription and DNAse treatment, on 1% Agarose gel. The four RNAs were obtained from a separated batch of in vitro transcription. GoI: gene of interest, T7: bacteriophage T7 promoter-derived sequence, EMCV-IRES: encephalomyocarditis virus internal ribosome entry site, UTR: untranslated region, His: histidine tag, GFP: green fluorescent protein.

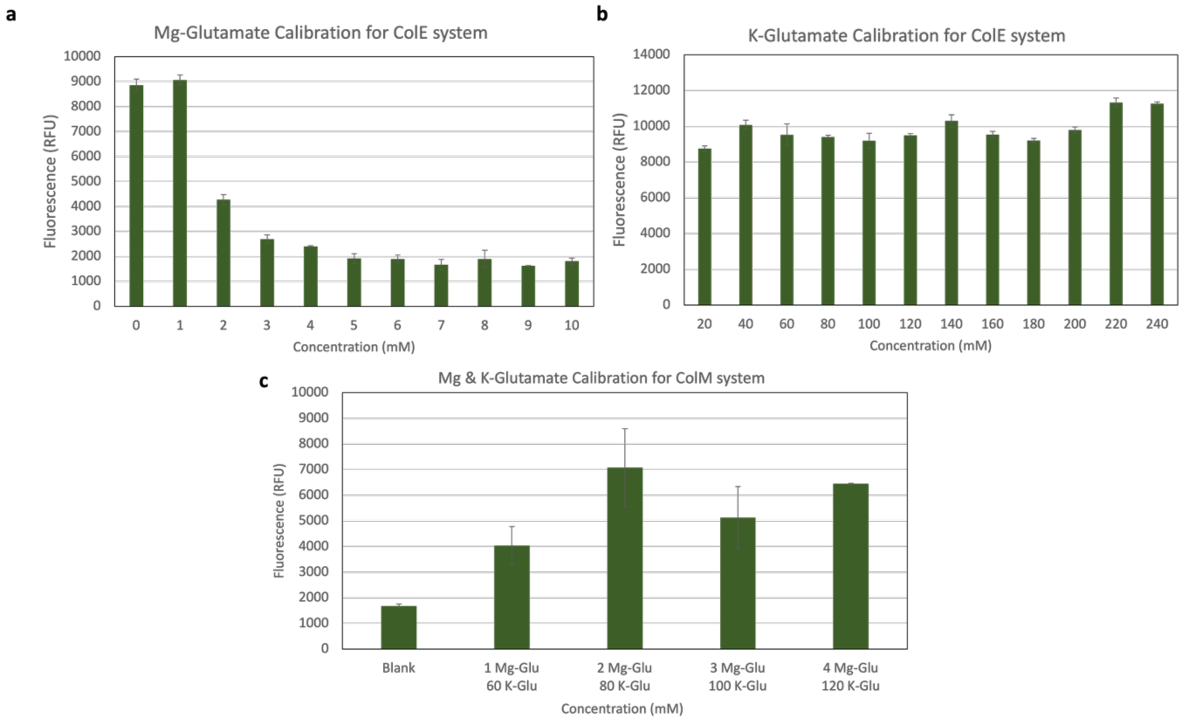

Figure S8: Preliminary calibration for Synthelis' cell-free lysate. The lysate must be calibrated in advance for Mg-glutamate and K-glutamate concentrations. Fluorescence here was produced using the DNA plasmid of P70a-deGFP. The overnight endpoint readings were normalized to blank samples (without DNA). Then we chose these concentrations of a) Mg-glutamate at 1 mM and b) K-glutamate at 220 mM for giving the highest production. These figures show the calibration for the Synthelis lysates batch used for colicin E1 and c) shows the calibration done for the Synthelis lysates batch used for colicin M.

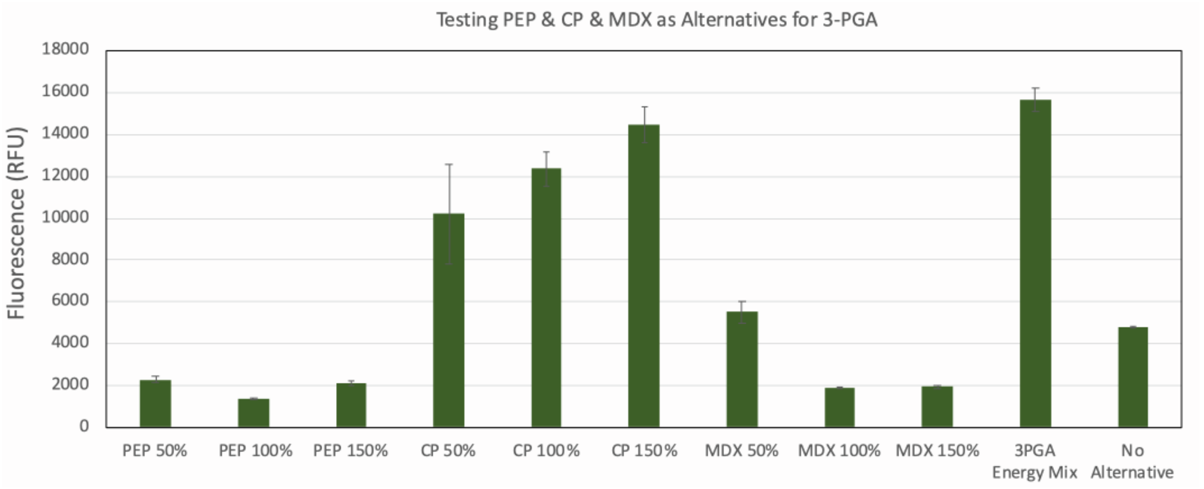

Figure S9: Testing energy source alternatives to 3-phosphoglyceric acid (3-PGA). Fluorescence was produced using the split-GFP colicin M DNA constructs (*GFP11-ColM* and *GFP1-10*). We tested three different energy components, which were phosphoenolpyruvate

(PEP), maltodextrin (MDX), and creatine phosphate (CP) for our proCFPS system. For each component, three different concentration ratios were tested. The 100% concentration references were 33 mM for PEP, 100 mM for CP (without creatine kinase), and 12 mg/mL for maltodextrin with 0.6 mg/mL of sodium hexametaphosphate (HMP). We finally chose to proceed with the CP as it showed similar performance to the energy mix containing 3-PGA.

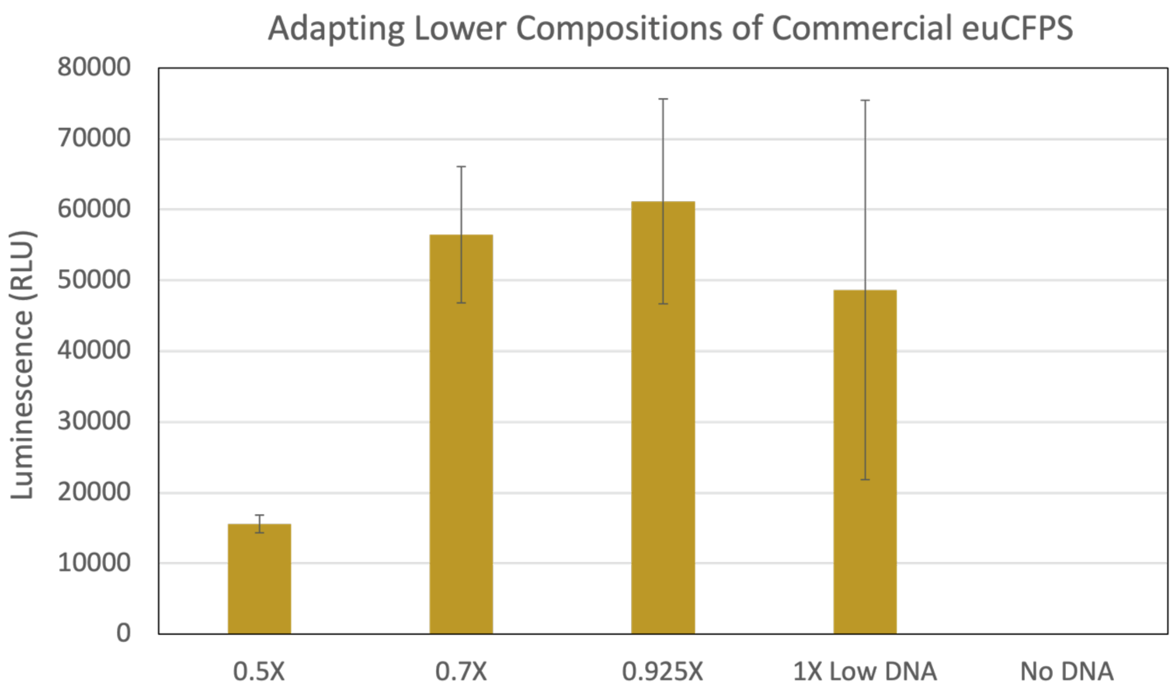

Components	DNA 7.1 nM	DNA 9.93 nM	DNA 13.13 nM	1X (DNA 11 nM)	No DNA
HeLa Lysate (2X)	500	700	925	1000	500
Access Prot (10X)	100	140	185	200	100
Reaction Mix (5X)	200	280	370	400	200
DNA (55nM)	258	361.2	477.3	400	0

Figure S10: Testing different lower ratios of the reference euCFPS commercial composition. The commercial euCFPS lysate was tested with different composition ratios to reach the recommended final DNA concentration starting from 55 nM stock concentration. The maximum composition we could reach was 0.925X of the original kit composition, which corresponds to 13.13 nM of final DNA concentration. All the reactions were done in 2µL by ECHO® while incubating for 1.5 hours at 30ºC in the Synergy HTX BioTek reader. Luminescence was performed using the Nano-Glo® HiBiT Lytic Detection system. The end-point readings (at gain 150) were normalized to the no DNA sample, and the error bars represent the standard deviation of 3 replicates performed within the same plate run. We chose the 0.5X composition as our new reference buffer for the active learning loops.

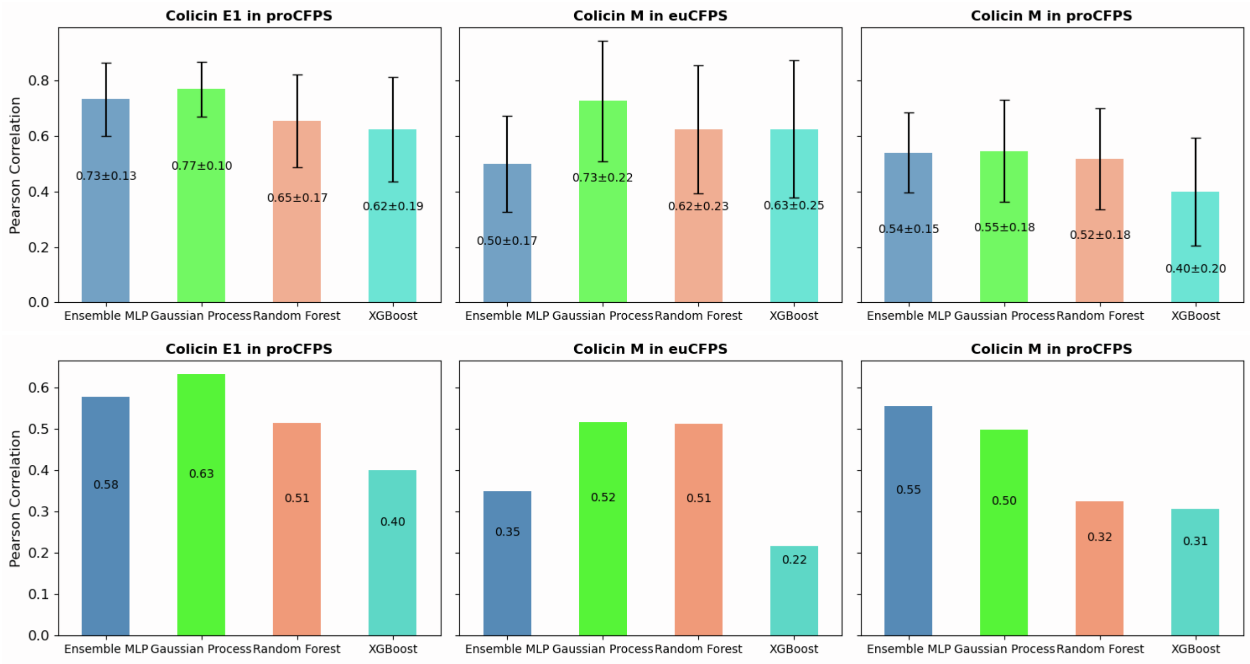

Figure S11: Preliminary Evaluation of Different Algorithms for Initiating Active Learning. This figure presents an initial evaluation of various machine learning architectures to determine the most suitable approach for initiating active learning. We compared different algorithms from the literature against the Gaussian process model to assess their relative performance.In the top row, we show the results from an initial test using the first dataset from three cell-free systems (Round 0). The dataset was randomly split into training (80%) and testing (20%) subsets, and this process was repeated 100 times to estimate the mean and standard deviation of the model performance.The bottom row presents a validation step, where the selected model's performance is assessed on real data obtained from the final round of active learning. The Gaussian process model was chosen due to its ability to perform effectively on small datasets. Moreover, it provides reliable uncertainty estimates, which are crucial for exploration in active learning frameworks. While ensemble-based multilayer perceptron (MLP) models and XGBoost are powerful machine learning approaches widely used in other studies, they are more susceptible to overfitting when trained on limited data. Their performance typically improves with larger datasets, making them less suitable for early-stage active learning applications.

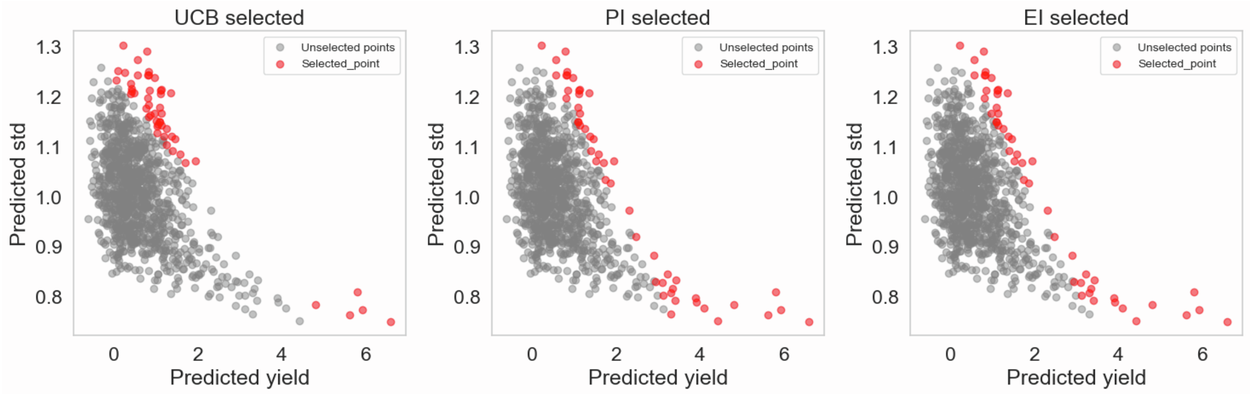

Figure S12: Comparison between different acquisition functions. Many acquisition functions can be interpreted within the framework of Bayesian decision theory, where they evaluate the expected loss associated with selecting a point x for function evaluation. The most

used acquisition functions are Upper Confidence Bound (UCB), Probability of Improvement (PI), and Expected Improvement (EI). In practice, UCB requires manually adjusting the balance between exploitation (predicted mean $\mu(x)$) and exploration (predictive standard deviation $\sigma(x)$), as they are on different scales of measurement. This adjustment adds one more tunable parameter, while in contrast, PI and EI do not require such adjustments and, in the context of our dataset, tend to select nearly identical data points. However, in theory EI considers both the magnitude and probability of improvement, making it a more robust choice as our acquisition function.

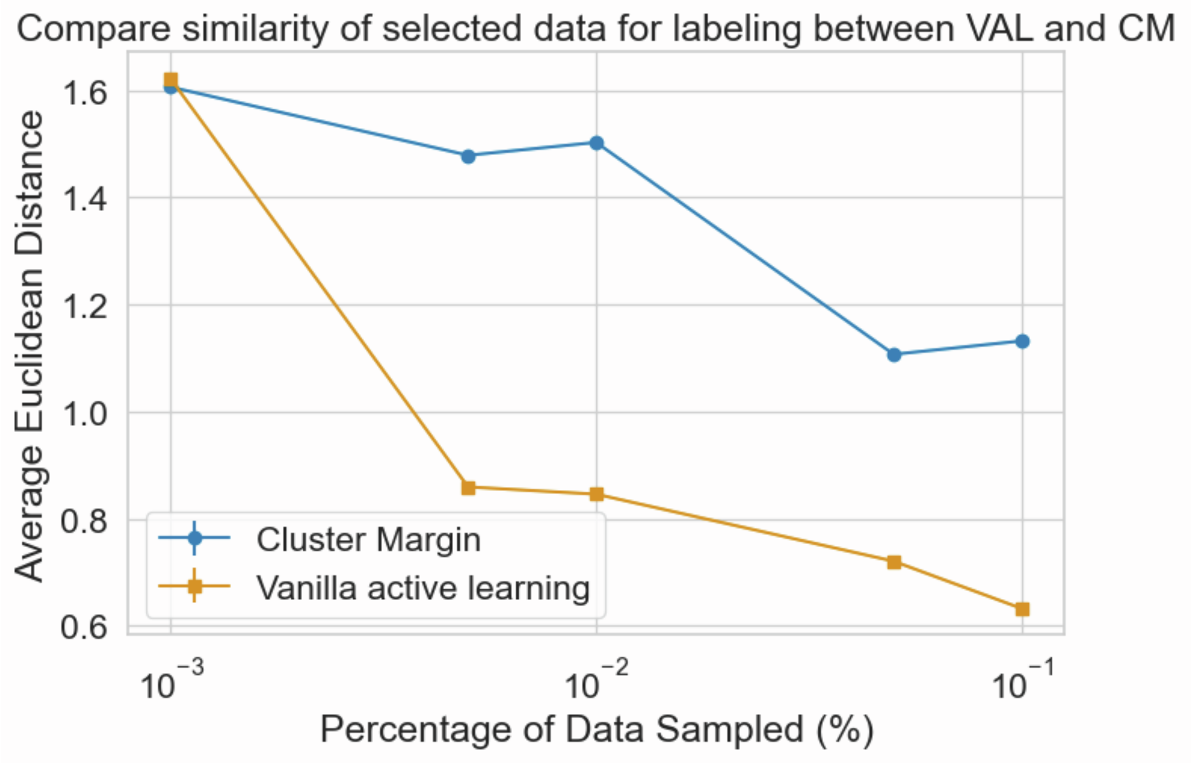

Figure S13: Effect of sampling ratio on average distance in chosen set (repeated 10 times). This figure illustrates the limitations of an exhaustive search in standard VAL. As the number of sampled data points increases across all possible selections while keeping the number of labeled points constant, the chosen points tend to become more similar to each other. This effect highlights a fundamental drawback of the Expected Improvement (EI) acquisition function in traditional optimization: it is designed to select a single point for labeling at each iteration and does not account for diversity among selected points. In contrast, the CM approach improves this by selecting points from different clusters sequentially, ensuring that the dataset remains diverse and that selected points are not overly concentrated in a specific region of the data space. This strategy helps mitigate redundancy and improves the representativeness of the labeled dataset and also the opportunity to sample a much bigger proportion of the pool while keeping the diversity.

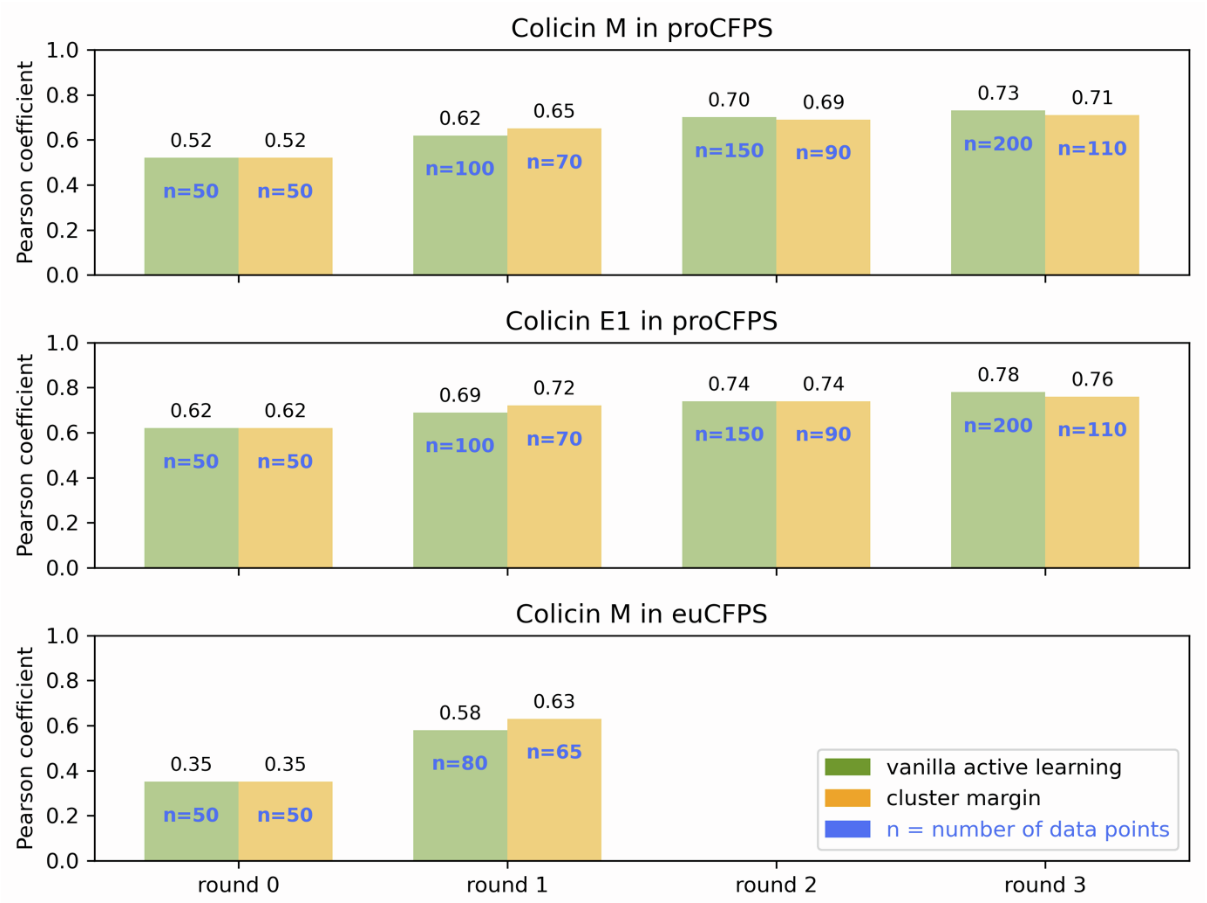

Figure S14: Compare model performance between VAL vs CM. Each diagram contains Pearson coefficients of both VAL and CM methods throughout each round. Starting with the same initial dataset, different training sets are selected at each round according to the respective methods. Consequently, two independent models are trained to suggest new data points at each round: 50 for VAL method (resp. 20 for CM method) new points for the proCFPS system and 30 (resp. 15) new points for the euCFPS system. For each system, we used data from the very last round as a test set and observed the Pearson correlation between model prediction and lab measurements at each round.

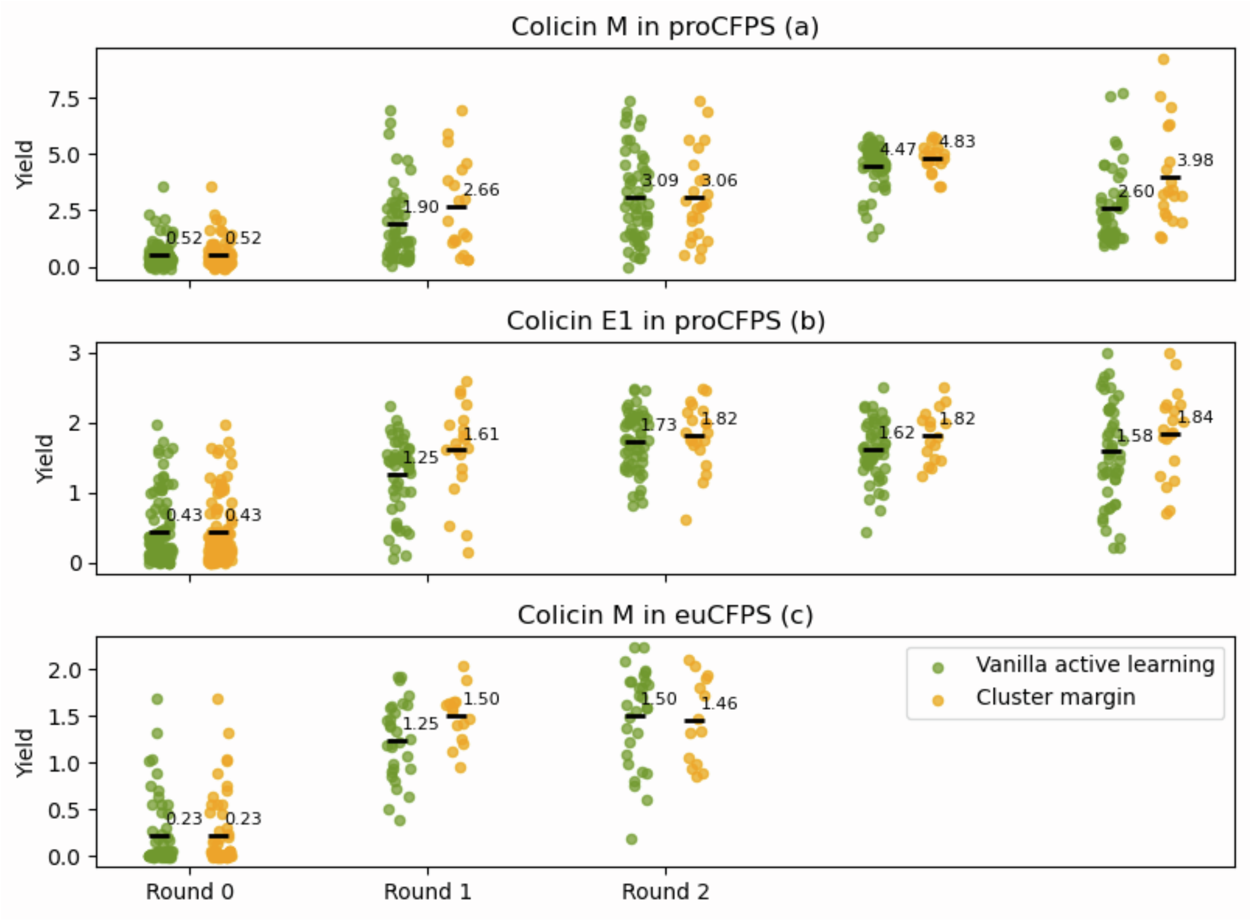

Figure S15: Compare yield achieved by VAL and CM. This figure extends Figure 3 from the main paper and presents the yield achieved at each step using both methods. By comparing the average yield across multiple repetitions of each experiment, we observe that there is no statistically significant difference in the highest yield obtained between VAL and CM. This result indicates that CM is also capable of identifying the highest yield, even with fewer data points or fewer experimental iterations.

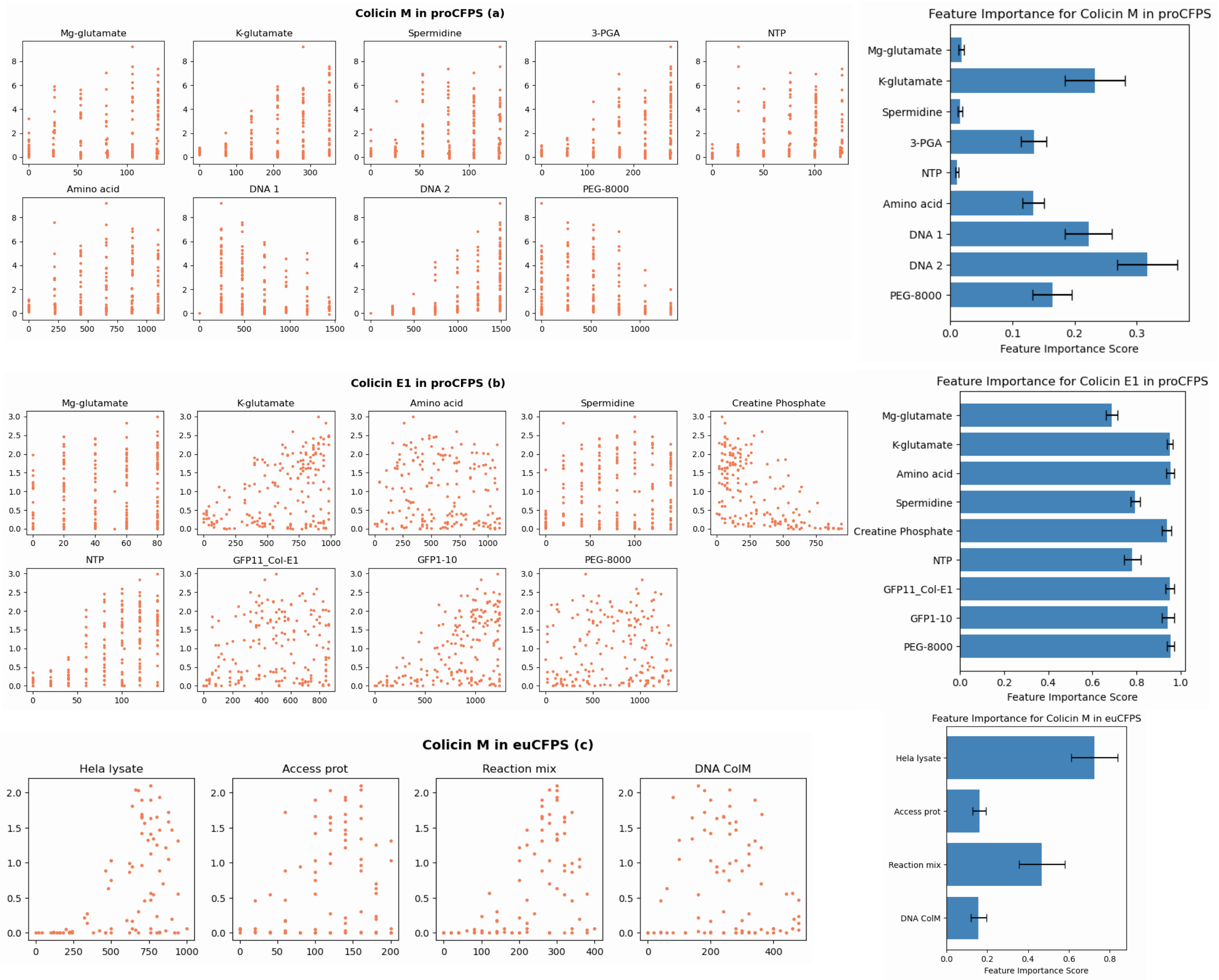

Figure S16: Interpreted model in each system. The figures present individual feature trends plotted against yield for each system, illustrating their impact on production. A horizontal bar plot ranks feature importance, indicating the significance of each compound in predicting yield—the higher the score, the more influential the feature in the model's predictions.

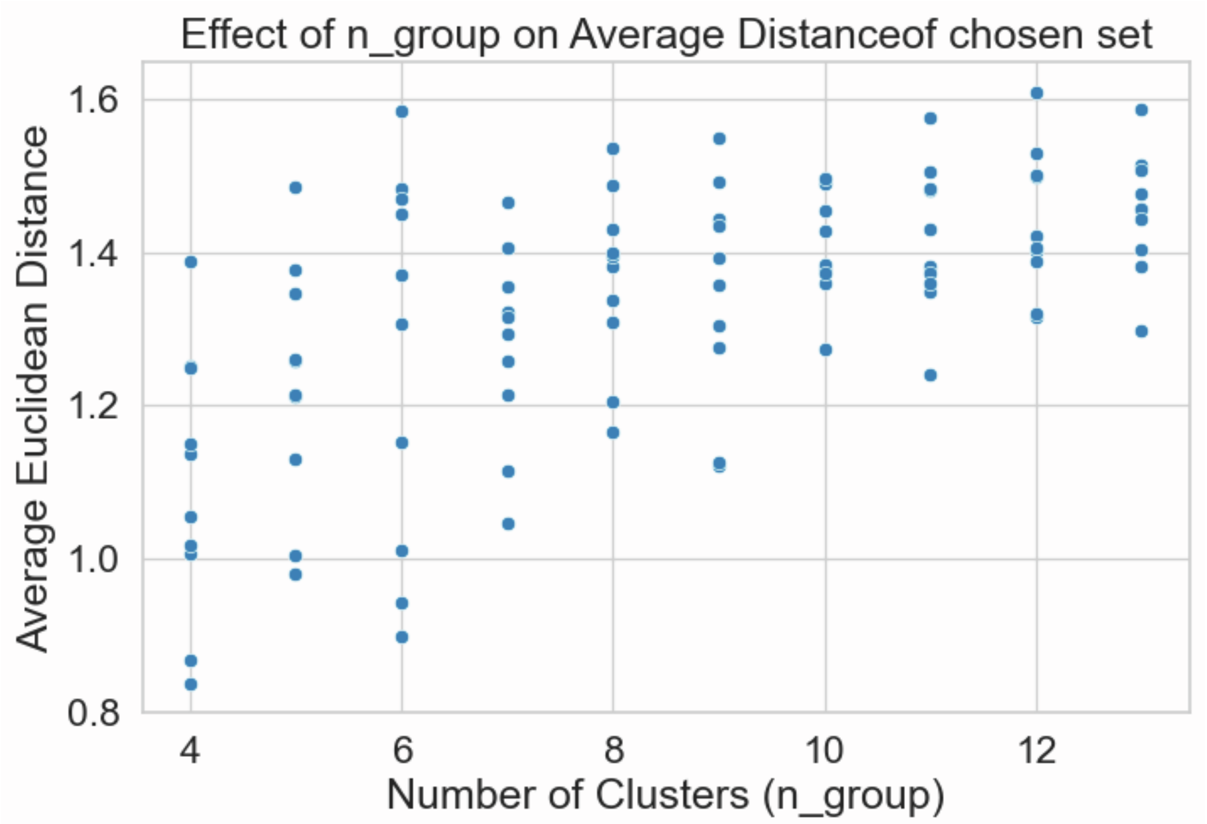

Figure S17: Effect of the Number of Clusters on the Average Similarity of the Chosen Set. This figure presents an example of selecting the number of clusters for the Cluster Margin (CM) method in the proCFPS system. The number of clusters is a crucial parameter in CM, as it directly influences the diversity of the selected points. If too few clusters are used, the chosen points may not sufficiently represent the dataset's variability. Conversely, increasing the number of clusters excessively does not necessarily enhance selection quality. While a higher number of clusters increases computational complexity, it does not lead to a significant rise in the average distance between chosen points. These findings suggest that an optimal number of clusters must be carefully chosen to balance dataset representativeness and computational efficiency.

Table S1. Maximum volumes of the components in the cell-free systems used. These maximum values define the search space of our system; they represent the volumes (nL) corresponding to the maximum concentrations, which is 125% of the proCFPS standard concentrations and 0.925X of euCFPS default kit concentrations. The maximum volume values for colicin M in proCFPS was automatically rounded up to the nearest multiple of 2.5, which is the minimum droplet volume transferred by ECHO®. While maximum volume values for colicin E1 in proCFPS and colicin M in euCFPS are manually rounded up to the nearest multiple of 20, which is the increment used.

proCFPS components ColM	Max Final Conc.(mM)	Max value (nL)	proCFPS components ColE1	Max Final Conc. (mM)	Max value (nL)	euCFPS components ColM	Max Final Conc. (X)	Max value (nL)
Mg-glutamate	2.5	131.3	Mg-glutamate	1.25	80	HeLa Lysate	0.925	940
K-glutamate	100	350	K-glutamate	275	980	Accessory proteins	0.925	200
Amino acids	1.875	1094	Amino acids	1.875	1100	Reaction mix	0.925	380
3-PGA	37.5	281.3	Creatine phosphate	150	940	HiBiT_ColM	0.925	480
NTPs	1.875	126.2	NTPs	1.875	140			
Spermidine	1.25	131.3	Spermidine	1.25	140			
GFP11_ColM	0.006	1434	GFP11_ColE1	0.005	860			
GFP1-10	0.006	1485	GFP1-10	0.005	1240			
PEG-8000	2.5	1312	PEG-8000	2.5	1320			

Table S2. Coding sequences of the Colicins M and E1 used in this study. The fully annotated plasmid sequences are available in the Materials Availability section.

DNA	Sequence
Colicin M	ATGGAAACCTTAACTGTTCATGCACCATCACCATCAACTAACTTACCAAGTTATGGCAA<br>TGGTGCATTTTCTCTTTCAGCACCGCATGTTCCTGGTGCTGGACCTCTTTTAGTCCAG<br>GTTGTTTATAGTTTTTTCCAGAGTCCAAACATGTGTCTTCAGGCTTTAACTCAACTTGA<br>GGATTACATCAAAAAACATGGGGCTAGCAACCCTCTCACATTGCAGATCATATCGACA<br>AATATTGGTTACTTCTGTAACGCCGACCGAAATCTGGTTCTTCACCCTGGAATAAGCG<br>TTTATGACGCTTACCACTTCTCAAAACCAGCGCCAAGTCAATATGACTATCGCTCAAT<br>GAATATGAAACAAATGAGCGGTAATGTCACTACACCAATTGTGGCGCTTGCTCACTAT<br>TTATGGGGTAATGGCGCTGAAAGGAGCGTTAATATCGCCAACATTGGTCTTAAAATTT<br>CCCCTATGAAAATTAATCAGATAAAAGACATTATAAAATCTGGTGTAGTAGGTACATTC<br>CCTGTTTCTACAAAGTTCACACATGCCACTGGTGATTATAATGTTATTACCGGTGCATA<br>TCTTGGTAATATCACACTGAAAACAGAAGGTACTTTAACTATCTCTGCCAATGGCTCCT<br>GGACTTACAATGGCGTTGTTCGTTCATATGATGATAAATACGATTTTAACGCCAGCAC<br>TCACCGTGGCGTCATCGGAGAGTCGCTCACAAGGCTCGGGGCGATGTTTTCTGGTAA<br>AGAGTACCAGATACTGCTTCCTGGTGAAATTCACATTAAAGAAAGTGGTAAGCGATAA
Colicin E1	ATGGAAACCGCGGTAGCGTACTATAAAGATGGTGTTCCTTATGATGATAAGGGACAG<br>GTAATTATTACTCTTTTGAATGGTACTCCTGACGGGAGTGGCTCTGGCGGCGGAGGT<br>GGAAAAGGAGGCAGTAAAAGTGAAAGTTCTGCAGCTATTCATGCAACTGCTAAATGGT<br>CTACTGCTCAATTAAAGAAAACACAGGCAGAGCAGGCTGCCCGGGCAAAAGCTGCAG<br>CGGAAGCACAGGCGAAAGCAAAGGCAAACAGGGATGCGCTGACTCAGCGCCTGAAG<br>GACATCGTGAATGAGGCTCTTCGTCACAATGCCTCACGTACGCCTTCAGCAACAGAG<br>CTTGCTCATGCTAATAATGCAGCTATGCAGGCGGAAGACGAGCGTTTGCGCCTTGCG<br>AAAGCAGAAGAAAAAGCCCGTAAAGAAGCGGAAGCAGCAGAAAAGGCTTTTCAGGAA<br>GCAGAACAACGACGTAAAGAGATTGAACGGGAGAAGGCTGAAACAGAACGCCAGTT<br>GAAACTGGCTGAAGCTGAAGAGAAACGACTGGCTGCATTGAGTGAAGAAGCTAAAGC<br>TGTTGAGATCGCCCAAAAAAAACTTTCTGCTGCACAATCTGAAGTGGTGAAAATGGAT<br>GGAGAGATTAAGACTCTCAATTCTCGTTTAAGCTCCAGTATCCATGCCCGTGATGCAG<br>AAATGAAAACGCTCGCTGGAAAACGAAATGAACTGGCTCAGGCATCCGCTAAATATAA<br>AGAACTGGATGAGCTGGTCAAAAAACTATCACCAAGAGCCAATGATCCGCTTCAGAA<br>CCGTCCTTTTTTTGAAGCAACCAGACGACGGGTTGGGGCCGGTAAGATTAGAGAAGA<br>AAAACAAAAACAGGTAACAGCATCAGAAACACGTATTAACCGGATAAATGCTGATATA<br>ACTCAGATCCAGAAGGCTATTTCTCAGGTCAGTAATAATCGTAATGCCGGTATCGCTC<br>GTGTTCATGAAGCTGAAGAAAATTTGAAAAAAGCACAGAATAATCTCCTTAATTCACAG<br>ATTAAGGATGCTGTTGATGCAACAGTTAGCTTTTATCAAACGCTGACTGAAAAATATG<br>GTGAAAAATATTCGAAAATGGCACAGGAACTTGCTGATAAGTCTAAAGGTAAGAAAAT<br>CGGCAATGTGAATGAAGCTCTCGCTGCTTTTGAAAAATACAAGGATGTTTTAAATAAG<br>AAATTCAGCAAAGCCGATCGTGATGCTATTTTTAATGCGTTGGCATCGGTGAAGTATG<br>ATGACTGGGCTAAACATTTAGATCAGTTTGCCAAGTACTTGAAGATTACGGGGCATGT<br>TTCTTTTGGATATGATGTGGTATCTGACATCCTAAAAATTAAGGATACAGGTGACTGGA<br>AGCCACTATTTCTTACATTAGAGAAGAAAGCTGCAGATGCAGGGGTGAGTTATGTTGT<br>TGCTTTACTTTTTAGCTTGCTTGCTGGAACTACATTAGGTATTTGGGGTATTGCTATTG<br>TTACAGGAATACTATGCTCCTATATTGATAAGAATAAACTTAATACTATAAATGAGGTG<br>TTAGGGATTTAA

Table S3. Primers used for PCR and the restriction enzymes for cloning the DNA fragments into the pIVEX vector.

Fragment	Primers used	For	Enzyme
Colicins (M and E1)	CcgtctcTTTAATACGACTCACTATAGGGAGACC<br>CcgtctcATCAGCAAAAAACCCCTCAAGACC	Fragment amplification	BsmBI
	GgaagacTTCTGAAAGGAGGAACTATATCCGG<br>AgaagacTATTAATTTCGCGGGATCGAGATC	pIVEX Backbone amplification	BbsI
GFP1-10	CcgtctcTATGTCTAAAGGTGAAGAACTGTTCACC<br>CcgtctcATCATTTTTCGTTCGGGTCTTTAG	Fragment amplification	BsmBI
	AgaagacAGACATGCGGCCTTCG<br>GgaagacTTATGATATCAAGATCCGGTAAGATCC	pIVEX Backbone amplification	BbsI
GFP11	CcgtctcTATGCGTGACCACATGG<br>CcgtctcATTATTTGTACAGTTCGTCCATACC	Fragment amplification	BsmBI
	AgaagacAGGCATGCGGCCTTCG<br>GgaagacTTATAATGATATCAAGATCCGGTAAGATCC	pIVEX Backbone amplification	BbsI
Primers used for adding GFP11 to the Colicin fragment (M or E1)			
GFP11-Colicins (M or E1)	GgaagacTTCTAGCATAACCCCTTGGGG<br>AgaagacAGTTTGTACAGTTCGTCCATACCG	Fragment amplification	BbsI
	AggtctcTCAAAAGGATCCGCATCGAAGG<br>AggtctcGctagTTATTGCTCAGCGG	Backbone amplification	BsaI

Table S4. Optimized buffer concentrations of the components in the cell-free systems used. The table presents both the standard and optimized concentrations of key buffer components used for both colicins in proCFPS and colicin M in euCFPS. For proCFPS, concentrations are shown in mM, where the standard values represent the ratio of 100% of the standard reference. For euCFPS, the standard buffer corresponds to 0.5X with its respective reagent volumes given in nL, so as for the optimized values. Blank entries indicate components not used in the respective formulation.

	ColM proCFPS		ColE1 proCFPS			ColM euCFPS	
Buffer	Standard (mM)	Optimized (mM)	Standard (mM)	Optimized (mM)	Buffer	Standard (nL)	Optimized (nL)
Mg-glutamate	2	2	1	1.5	HeLa Lysate	500	700
K-glutamate	80	80	220	257	Accessory proteins	100	160
Amino acids	1.5	1.13	1.5	0.583	Reaction mix	200	240
3-PGA	30	37.67	___	___	HiBiT_ColM	258	320
Creatine phosphate	___	___	100	6.4			
NTPs	1.5	0.37	1.5	2.08			
Spermidine	1	1.26	1	0.952			
GFP11_ColM	0.006	0.001	___	___			
GFP11_ColE1	___	___	0.005	0.0029			
GFP1-10	0.006	0.006	0.005	0.00493			
PEG-8000	2	0	2	0.8			

Algorithm: Calibrate Second Batch Data to First Batch Level

Input:

- $S = \{x, yield\}$,
- $S_{ref} = \{x, yield_{mean\,0}\}$ is the level where you want to align with (initial batch or calibrated previous batch),
- $R^2{}_{limit}$ (default = 0.8),
- (x_{Ref+}, x_{Ref-})
- n (default 10) is the number of reference points selected for the next round.

Output:

- $S' = \{x, yield'_{mean}\}$, with $yield'_{mean}$ is averaged and calibrated yield,
- R as reference points to incorporated with AL results for calibration of next round

Notations

- $X = \{x \mid x\ is\ a\ component\ combination\}$ is the set of component combinations
- $Y = \{\{y \mid y\ is\ a\ yield\ value\}\}$ is the set of each measured yields set
- $n = |X| = |Y|$ is the number of combinations tested
- $S = \{(x_i, Y_i) \mid x \in X, y \in Y, 1 \le i \le n\}$ is the set of component combinations and their yields values
- $S_{cal} = \{(x_i, y_{cal\,i}) \mid x \in X, 1 \le i \le n\}$ is the set of component combinations and their average calibrated yield value
- S_{ref} is the set of component combinations and their average calibrated yield value of the reference batch to align with
- $R \subseteq S_{cal}$ is the set of reference points

Steps

1. **Preprocessing**
   - Compute yields average for each component combination. Let's note $S' = \{X, Y'_{mean}\}$ with $Y'_{mean} = \{y'_{mean} \mid y'_{mean} = \underline{Y'_i}, 1 \le i \le n\}$
2. **Identification and Alignment of Repeated Experiments**
   - $R^2 \longleftarrow 0$
   - $WHILE\ R^2 \le R^2{}_{limit}$
     1. Fit an OLS regression to each pair of $\{(y_i, y_{ref\,i}) \mid x_i = x_{ref\,i}, (x_i, y_i) \in R, 1 \le i \le n\}$. We then obtain a and b of the formula $y_{ref\,i} = a * y_i + b$, and R^2 value.
     2. Calculate the Cook's distance of each data pair
     3. Remove the pair with highest distance (remove outliers) (less than 30% of points)
3. **Calibration**
   - Transform all $\text{yield}_{\text{mean}}$ into $\text{yield'}_{\text{mean}}$ by the newest OLS regression line (a*yield + b) to calibrate all experiments in the second batch to the first batch level.

4. **Picking of reference points for next batch** *(change reference points in order to cover all the range of yield values)*

   - $R' \longleftarrow \emptyset$
   - $R' \leftarrow R' \cup \{(x_{Ref+}, yield'_{Ref+})\} \cup \{(x_{Ref-}, yield'_{Ref-})\} \cup \{(x, yield'_{max})\}$
   - $R' \longleftarrow R' \cup \{x \in_R S\}_{n-3}$
   - Return $(S_{cal} \setminus R, R')$

To compare different buffer compositions, we calculated the yield of the AMP production based on the fluorescence values as the following:

$$Yield_i = \frac{Fluo_i - Autofluo_i}{Fluo_{ref} - Autofluo_i}$$
